# Spatiotemporal control of myoblast identity drives muscle diversity in the *Drosophila* leg

**DOI:** 10.1126/sciadv.aed0910

**Published:** 2026-07-01

**Authors:** Camille Guillermin, Violaine Tribollet, Mathilde Bouchet, Anne Laurencon, Dan Zhou, Sergio Sarnataro, Laurent Gilquin, Isabelle Stevant, Guillaume Marcy, Emeric Texeraud, Yad Ghavi-Helm, Benjamin Gillet, Sandrine Hughes, Samantha Vonau, Jonathan Enriquez

**Affiliations:** ^1^ Institut de Génomique Fonctionnelle de Lyon, ENS de Lyon, CNRS, Université Lyon 1, Lyon 69007, France.; ^2^ Université Claude Bernard Lyon 1, Bioinformatic Platform of the Labex Cortex, Lyon 69008, France.

## Abstract

Skeletal muscles exhibit notable morphological diversity, yet the developmental mechanisms specifying distinct identities are unclear. Using *Drosophila* leg muscles, we show that naïve mesodermal precursors undergo stepwise specification orchestrated by epithelial morphogens. Wg/Wnt1 and Dpp/BMP first restrict multipotent precursors into proximal and distal lineages. Within the distal lineage, successive fate bifurcations generate distinct muscle subtypes and a separate nonmuscle lineage of neuronal lamella cells. Focusing on one lineage, we demonstrate that Wg and Dpp act again to control the spatiotemporal deployment of transcription factors, ensuring that groups of myoblasts destined to fuse to produce a specific muscle share a coordinated transcriptional identity. Thus, epithelial morphogens not only pattern the epithelium but also synchronize myoblast specification, enabling the emergence of diverse muscles from syncytial fibers. Our findings provide a framework for the developmental and evolutionary origins of appendicular muscles and may help explain the selective vulnerability of specific muscles in muscular dystrophies.

## Teaser

This study reveals how muscle diversity arises in flies, enabling the complex movements that drive animal behavior.

## INTRODUCTION

What sets muscles apart from other organs is that, while they all share the property of contractility, they exhibit remarkable morphological diversity. This diversity underlies a wide range of behaviors across the animal kingdom, from feeding and mating to highly refined tasks such as writing. A notable example comes from appendicular muscles, which power locomotor behaviors in many species. Human limbs contain around 25 distinct muscles (*1*), a number comparable to those in *Drosophila* legs, enabling appendages in both organisms to move in all directions of space (*2*, *3*).

In most organs, cellular diversity is achieved by specifying the identities of individual cells. An extreme example is found in the *Drosophila* nervous system, where nearly every neuron acquires a unique identity (*4*, *5*). Muscles, however, are unusual: They are syncytial organs, formed by the fusion of many myocytes into multinucleated fibers (*6*). This syncytial nature means that identity must be coordinated not only at the level of individual cells but also across populations of cells that will later fuse.

In vertebrates, muscle progenitor cells (MPCs) arise from the hypaxial dermomyotome, migrate into the limb bud (*7*), proliferate, and then differentiate into myocytes that fuse to multinucleated fibers (*8*). According to the prevailing model, MPCs are naïve: They contribute passively to muscle morphogenesis by following a prepattern established by connective tissue (*8*–*11*). Yet an alternative possibility is that myoblasts actively acquire distinct fates through gene regulatory networks operating in parallel with the general myogenic program (*12*–*17*).

*Drosophila* provides a powerful comparative model, as leg muscle architecture and development closely mirror vertebrate limb muscles. Adult muscle precursors (AMPs) colonize the leg disc, proliferate, and later fuse into multifiber muscles (*18*). This system raises a central question: Do AMPs passively build unique muscles, or do they follow unique specification programs, influenced by extrinsic cues, to generate distinct morphologies? Notably, epithelial signals have been implicated in patterning indirect and direct flight muscles, generating two muscle populations with distinct sarcomeric organization and contractile properties (*19*–*21*). This raises the possibility that epithelial-derived morphogens may also act instructively on leg muscle precursors, guiding the transition from a naïve state to defined muscle lineages before the fusion process.

Here, we combine lineage tracing, single-cell transcriptomics, genetic perturbations, and spatial imaging to reconstruct the developmental trajectories of AMPs in the *Drosophila* leg. We show that AMPs progressively acquire distinct temporal and spatial identities through a multistep process that gives rise to diverse muscle lineages as well as a neuronal lamella lineage before fusion. On this basis, we propose to rename AMPs as mesodermal precursors (MPs), reflecting their ability to generate both muscle and nonmuscle lineages. This diversification is coordinated by positional cues from the epithelium, which ensure cross-tissue developmental integration and specify groups of myoblasts that share a common transcriptional identity and will ultimately fuse to build a single muscle. Together, these results provide a framework for understanding the developmental and evolutionary origins of limb muscle patterning.

## RESULTS

### Diversity of muscle morphologies in the T1 thoracic segment

**Fig. 1.**
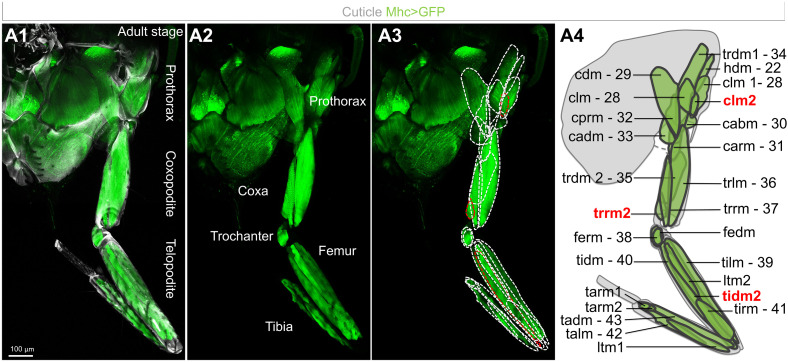
Adult muscle diversity in the *Drosophila* foreleg. (**A1** to **A4**) Maximum projection of adult leg muscles genetically labeled with *UAS-mCD8::GFP* (green) under the control of *MHC-GAL4* [(A1) to (A3)], and a schematic representation of all adult muscles (A4). Muscle nomenclature follows (*2*, *3*): m, muscle; hd, head dorsal; c, coxa; tr, trochanter; fe, femur; ti, tibia; ta, tarsus; l, levator; d, depressor; ab, abductor; ar, anterior rotator; pr, posterior rotator; ad, adductor; r, reductor; lt, long tendon; or A. Miller’s nomenclature (*56*): numbers. See also fig. S1 and S2 and movie S1.

The muscle architecture of the T1 leg has been documented in previous studies (*2*, *3*). Here, we reexamined the architecture of leg muscles, including those in the thorax responsible for prothoracic leg movement, using a new protocol (see Materials and Methods). We identified three previously undocumented muscles: the coxa levator muscle 2 (*clm2*) in the prothorax, the trochanter reductor muscle 2 (*trrm2*) in the coxa, and the tibial depressor muscle 2 (*tidm2*) in the femur (Fig. 1, figs. S1 and S2, and movie S1).

### Description of mesodermal precursor and myoblast behavior during development

**Fig. 2.**
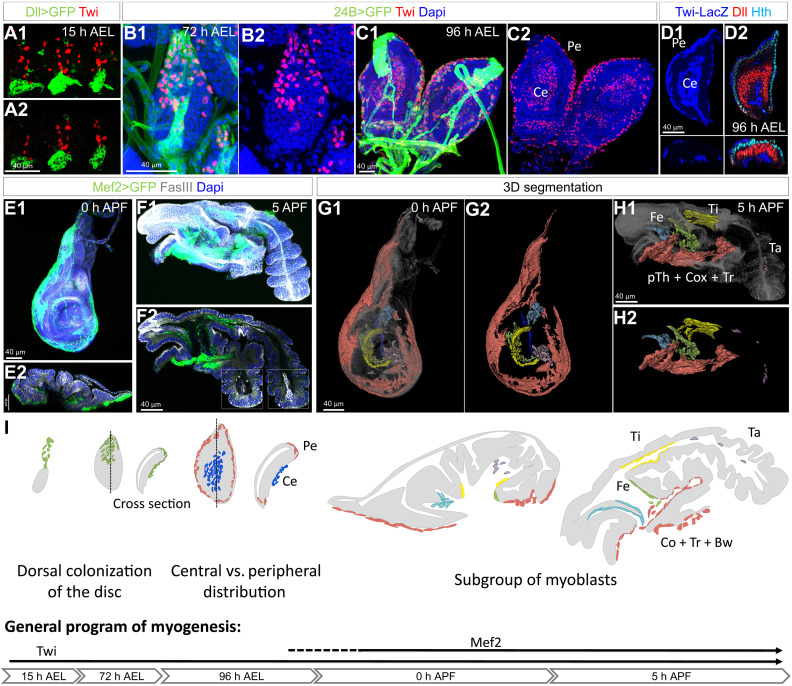
The progressive spatial organization of myoblasts into specific clusters. (**A1** and **A2**) Maximum projection (A1) and confocal section (A2) of three thoracic hemisegments of an embryo at 15 hours after egg laying (AEL). Epithelial cells of the leg disc are genetically labeled with *UAS-mCD8::GFP* (green) under the control of *Dll-GAL4*, and myoblasts immunolabeled with anti-Twist (red). (**B1** and **B2**) Maximum projection of a T1 leg disc at 72 hours AEL; myoblasts labeled with *UAS-mCD8::GFP* (green) under the control of *24B-GAL4* and stained with anti-Twist (red); nuclei are counterstained with 4′,6-diamidino-2-phenylindole (DAPI; blue) (**C1** and **C2**) Same as (B1) and (B2), but at 96 hours AEL. (**D1** and **D2**) T1 leg disc at 96 hours AEL carrying *twi-LacZ* (blue): maximum projection [(D1), top], confocal section [(D2), top], and cross sections (bottom). Epithelial cells are stained with anti-Dll (red) and anti-Hth (cyan). Note: *twi-LacZ*^+^ myoblasts are localized centrally (Ce) beneath Dll^+^ epithelial cells, and peripherally (Pe) adjacent to Hth^+^ epithelial cells. (**E1** and **E2**) T1 discs during early pupal stages. Myoblasts are genetically labeled with *UAS-mCD8::GFP* (green) under the control of *Mef2-GAL4*, stained with anti-FasIII (gray), and nuclei counterstained with DAPI (blue). (E1) and (**F1**) Maximum projections; (E2) and (**F2**) Cross sections. The dashed boxes in (F2) highlight the weak GFP expression in the tarsus (asterisks), visible in a different focal plane. (**G1** to **H2**) Three-dimensional (3D) segmentation of myoblasts. pTh, prothorax; Cox, coxa; Tr, trochanter; Fe, femur; Ti, tibia; Ta, tarsus. The 3D movie of these images is presented in movie S2. (**I**) Top: Schematic of the coordinate development of the leg myoblasts and the epithelium. Bottom: The schematic of the general program of myogenesis. See also fig. S3. h, hours.

In our quest to understand how muscle diversity arises during development, we conducted a detailed analysis of AMP behavior, commonly called myoblasts, as they actively divide in the leg disc (Fig. 2).

Our study begins at the late embryonic stage, when the epithelial cells of the leg disc have evaginated from the ectoderm [stage 16, 15 hours after egg laying (AEL)]. At this stage, ~20 AMPs (Twi^+^) are closely associated with the dorsal region of epithelial precursors of the leg discs (Dll-GFP^+^) (Fig. 2, A1 and A2, and fig. S3A). As development progresses, by 72 hours AEL, the number of myoblasts increases to around 50. At this stage, myoblasts also begin to colonize the dorsal region of the disc (Fig. 2, B1 and B2, and fig. S3A). By 96 hours AEL, the number of myoblasts increases markedly to ~450, fully colonizing the leg imaginal disc and forming two distinct myoblast clusters: central and peripheral (Fig. 2, C1 to D2, and fig. S3A).

During late larval stages, Mef2 expression is initiated in a subset of Twi^+^ cells, predominantly at the periphery of the leg disc (fig. S3B1). Notably, at this stage, a distinct cluster of Twi^+^ cells is localized within the presumptive tarsal territory, despite the absence of muscle formation in this region (fig. S3, B2 and B3). By 0 hours after pupa formation (APF), all Twi^+^ cells express *Mef2>GFP* (fig. S3C1). However, Twi^+^ cells located in the tarsus presumptive territory show a marked reduction in Twist protein levels and exhibit only very low levels of *Mef2>GFP* expression (fig. S3, C2 and C3). By 5 hours APF, the tarsal cluster displays either very low (Fig. 2, F1 and F2) or no detectable *Mef2>GFP* signal, accompanied by a complete loss of detectable Twist protein (fig. S3, D1 to D3), suggesting that these cells are specified to a nonmuscle lineage.

At the beginning of the metamorphosis (0 hours APF), the central myoblast cluster subdivides into four smaller clusters, each localizing to specific presumptive territories: two in the femur, one in the tibia, and one in the tarsus. Meanwhile, the coxa, trochanter, and prothoracic segments remain unevaginated, with myoblasts in these regions organized into two concentric rings (Fig. 2, E1, E2, G1, and G2; and movie S2). By 5 hours APF, evagination occurs in the tarsal and tibial segments, accompanied by partial protrusion of the femoral segment, resulting in the internalization of the central myoblast clusters (Fig. 2, F1, F2, H1, and H2).

In summary, myoblasts colonize the disc dorsally, initially organizing into central and peripheral groups. The central cluster subsequently subdivides into four smaller clusters, suggesting that this spatial organization may prefigure the future arrangement of muscles (Fig. 2I). This spatial pattern of behavior before fusion closely resembles that observed in vertebrate limb muscle precursors (*8*–*11*).

### Myoblasts are progressively committed to producing specific muscles

**Fig. 3.**
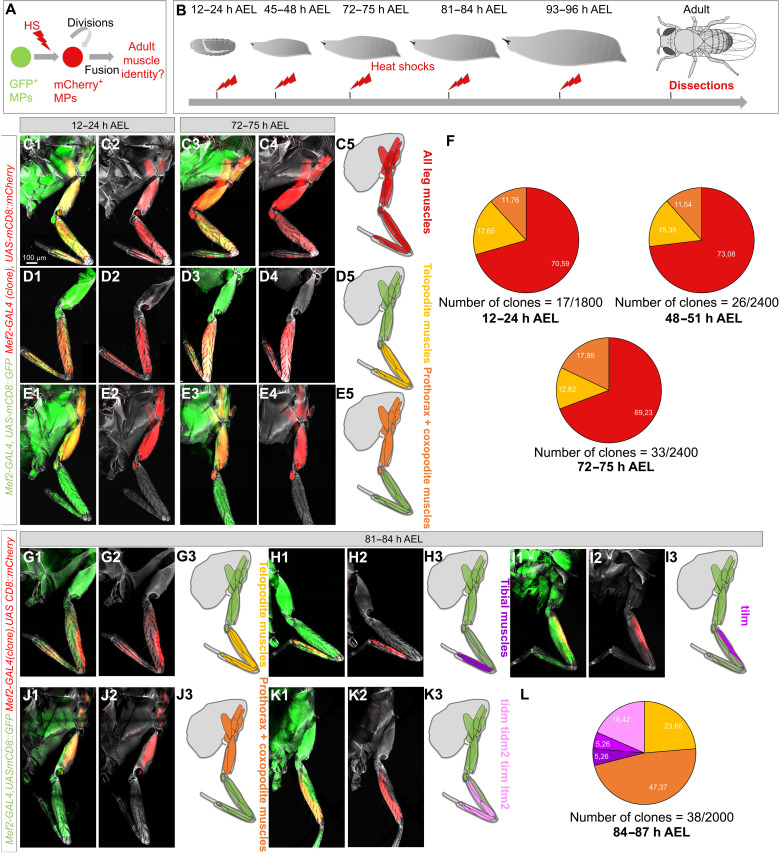
Random lineage tracing of myoblasts. (**A**) Schematic of the clonal induction of mCherry in myoblasts. (**B**) Schematic of the timing of the clonal induction of mCherry in myoblasts. (**C1** to **E5**) Maximum projection and schematic of the T1 leg and prothorax where all muscles are labeled with *UAS-mCD8::GFP* (green) under the control of *Mef2-GAL4* showing the expression of mCherry in muscle after its induction in myoblasts at 12 to 24 hours or at 72 to 75 hours AEL. The expression of the mCherry is color coded in the schematics of the leg. Note: We used the FlyBow1.1 system to induce randomly myoblast clones (see Materials and Methods). (**F**) Pie chart showing the portion of the three types of clones found after mCherry induction at 84 to 87 hours AEL. (**G** to **L**) Confocal images and corresponding schematics of the T1 leg where clones’ induction in myoblasts is triggered at 81 to 84 hours AEL. mCherry expression is color coded in the schematics, and the respective proportions are presented on a pie chart. *tilm*, tibia levator muscle; *tidm*, tibial depressor muscle; *ltm2*, long tendon muscle 2. h, hours.

To assess whether myoblasts are specified to produce distinct muscles before fusion, we generated clones at different developmental stages (Fig. 3, A and B). Previous studies (*3*) suggested that, during early larval stages, myoblasts have the potential to generate all prothoracic muscles (the legs were not analyzed as they had been removed in the experiment).

To determine whether myoblasts are specified before fusion, we first generated mCherry^+^ clones during the second half of embryogenesis, 12 to 24 hours AEL. Among 1800 examined flies, we identified 17 clones in the prothoracic segment. The majority of these clones (70%, *n* = 12 of 17) labeled all leg muscles across all leg segments, including the prothorax, a proportion consistent with our observations in the mesothoracic and metathoracic segments, although not detailed here (Fig. 3, C1 to C5 and F).

In a subset of cases (*n* = 4 of 12), clones extended into the contralateral hemisegment, including the leg, an occurrence previously reported in the thorax (*3*). Notably, in these instances, muscle labeling intensity was consistently weaker on one side. Given the rarity of such clone events and their absence in the mesothoracic and metathoracic segments, it is unlikely that these observations result from two independent clone events. Instead, we hypothesize that myoblasts migrate to the contralateral leg disc, a phenomenon that may occur specifically in the prothoracic segment due to physical contact between the T1 leg discs. These findings, where clones label all muscles in the prothoracic segment and can cross the body midline, strongly suggest that MPs are naïve before colonizing the leg disc.

In addition, we identified two other distinct myoblast clone types: one labeling muscle in the femur and tibia (telopodite) (18%, *n* = 3 of 17) (Fig. 3, D1, D2, D5, and F), and another labeling muscles in the trochanter, coxa (coxopodite), and prothorax (12%, *n* = 2 of 17) (Fig. 3, E1, E2, E5, and F). These findings raise two possible hypotheses: (i) Myoblasts begin to be specified into these two muscle subpopulations during embryogenesis, suggesting that most myoblasts remain uncommitted; or (ii) myoblasts specification occurs at later developmental stages.

To distinguish between these scenarios, we generated myoblasts clones during larval stages. Clones induced at 48 hours AEL (*n* = 26 of 2400) (Fig. 3F) and 72 hours AEL (*n* = 33 of 2400) (Fig. 3, C3 to C5, D3 to D5, and E3 to E5) exhibited similar results to embryonically induced clones. However, when clones were induced at 84 hours AEL (*n* = 38 of 2000), we no longer observed clones labeling all muscles (Fig. 3, G1 to L). Instead, clones predominantly labeled either the tibia and femur (Fig. 3, G1 to G3 and L) or the trochanter, coxa, and prothorax (Fig. 3, J1 to J3 and L). Intriguingly, at these later time points, we also identified clones labeling only a subset of muscles within the femur or tibia: (i) clones labeling random subsets of tibial muscles (Fig. 3, H1 to H3 and L); (ii) clones labeling the tibial levator muscle (*tilm*) in the femur (Fig. 3, I1 to I3 and L); and (iii) clones labeling the tibial depressor muscle (*tidm*), the *tidm2*, the tibial repressor muscle (*tirm*), and the long tendon muscle 2 (*ltm2*) in the femur (Fig. 3, K1 to K3 and L).

Collectively, these results indicate that myoblasts enter the disc in an unspecified state and subsequently specify into two populations: one producing distal muscles (tibia and femur) and the other producing proximal muscles (prothorax, coxa, and trochanter) after 84 hours AEL. These two populations may correspond to MPs localized in the disc center versus those at the periphery. Furthermore, the presence of subpopulation-specific clones at 84 hours AEL suggests that myoblasts gradually specify to generate both muscle subpopulations and individual muscles over time. Of course, another possibility is that these small clones result from reduced division of myoblasts after these stages; however, the stereotypy of clones consistently labeling a single muscle or a specific subpopulation of muscles instead suggests that myoblasts undergo specification through distinct, stereotyped lineages.

### The initial step in muscle specification involves defining distal versus proximal muscle lineages

**Fig. 4.**
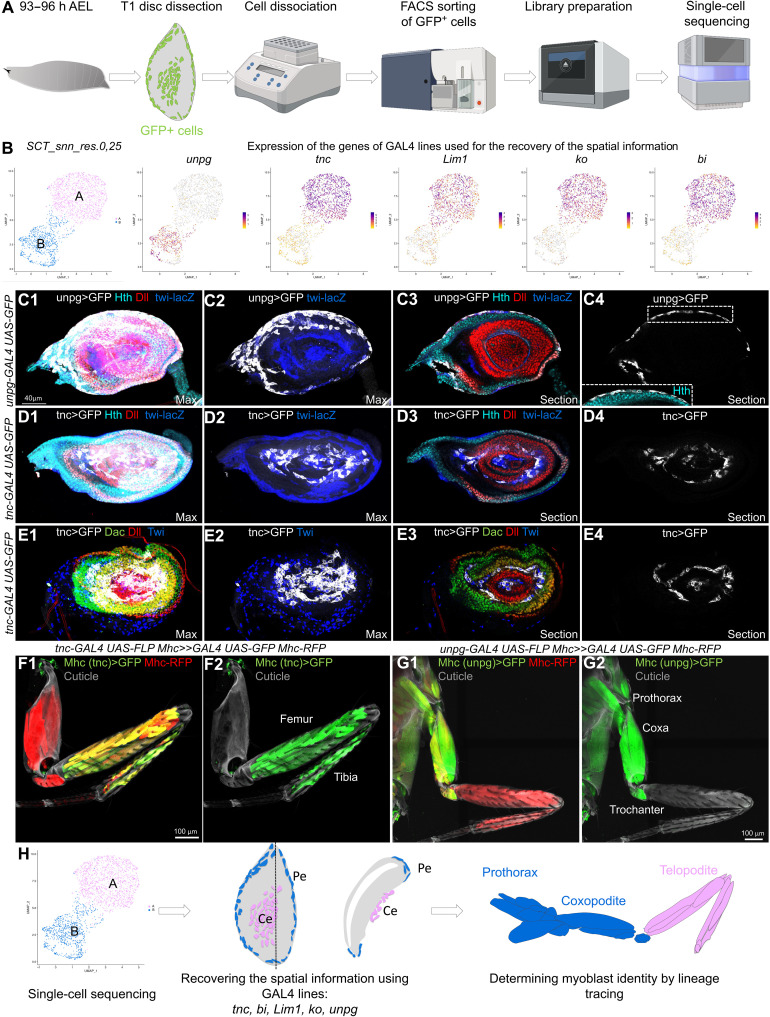
Two spatially and transcriptionally distinct populations of myoblasts give rise to proximal and distal muscles. (**A**) Schematic of the scRNA-seq workflow. (**B**) UMAPs showing two myoblasts clusters A and B (left) and the expression of spatial identity genes used in (C1) to (E4) (right). (**C1** to **E4**) Leg discs (at 96 hours AEL) from *twi-lacZ* (blue) larvae genetically labeled with *UAS-mCD8::GFP* under the control of *unpg-GAL4* [(C1) to C4)] or *tnc-GAL4* [(D1) to (E4)], and immunostained with anti-Dll (red) and anti-Hth (cyan) [(C1) to (D4)], or with anti-Dll (red) and anti-Dac (green) [(E1) to (E4)]. The dashed boxes in (C4) highlight the expression of Hth in peripheral unpg-GFP^+^ myoblasts. (**F1** to **G2**) T1 legs labeled with *Mhc-RFP* (red), showing GFP expression in distal and proximal muscles when the lineage tracing system is activated in myoblasts using *tnc-GAL4* [(F1) and (F2)] or *unpg-GAL4* [(G1) and (G2)], respectively. (**H**) Schematic summarizing the workflow used to determine the spatial position and identity of myoblast clusters. Pe, periphery; Ce, central. h, hours.

We began by investigating the first step of specification, proximal versus distal, by conducting single-cell RNA sequencing (scRNA-seq) of green fluorescent protein (GFP)^+^ myoblasts at 96 hours AEL (Fig. 4A) and unveiled two distinct cell clusters (clusters 0 and 1) (Fig. 4B).

To recapture the spatial information lost during fluorescence-activated cell sorting (FACS), we used GFP expression under the control of enhancer or gene trap lines specifically active in one or the other cluster. In parallel, we labeled the peripheral part of the epithelium, presumptive territory of the prothorax and the coxopodite (coxa and trochanter), and the central part of the epithelium, presumptive territory of the telopodite (femur and tibia) with Hth and Dll/Dac, respectively, to characterize the position of the two myoblasts clusters relative to the epithelium (Fig. 4, C1 to E4). We observed that myoblasts of cluster A labeled with GFP (*tnc*, *bi*, *Lim1*, or *ko-GAL4* lines) are located at the disc’s center (Dll^+^ and Dac^+^), whereas GFP^+^ MPs of cluster B (*unpg-GAL4* line) are localized at the periphery of the disc (Hth^+^) (Fig. 4, C1 to E4, and fig. S4, A1 to E3).

We then used a lineage muscle tracing system to determine the destiny of both myoblast populations until the adult stage. When we activated the lineage muscle tracing system using GAL4 under the control of *tnc*, *bi*, or *ko* (cluster A), GFP expression was observed in tibial and femoral muscles (Fig. 4, F1 and F2; and fig. S4, F to G3). Conversely, activation of the lineage muscle tracing system with *unpg-GAL4* (cluster B) resulted in GFP expression in muscles of the prothorax, coxa, and trochanter (Fig. 4, G1 and G2).

Our clonal analysis, combined with scRNA-seq, revealed that, during larval stages, MPs lose their potential to generate all muscle types and instead become specified into two distinct populations: one localized at the center and the other at the periphery of the disc. These populations give rise to distal and proximal muscles, respectively (Fig. 4H). The spatial arrangement of these populations prefigures the future positioning of distal and proximal muscles more than 48 hours before the fusion process.

### Myoblasts are progressively specified to generate unique muscles

**Fig. 5.**
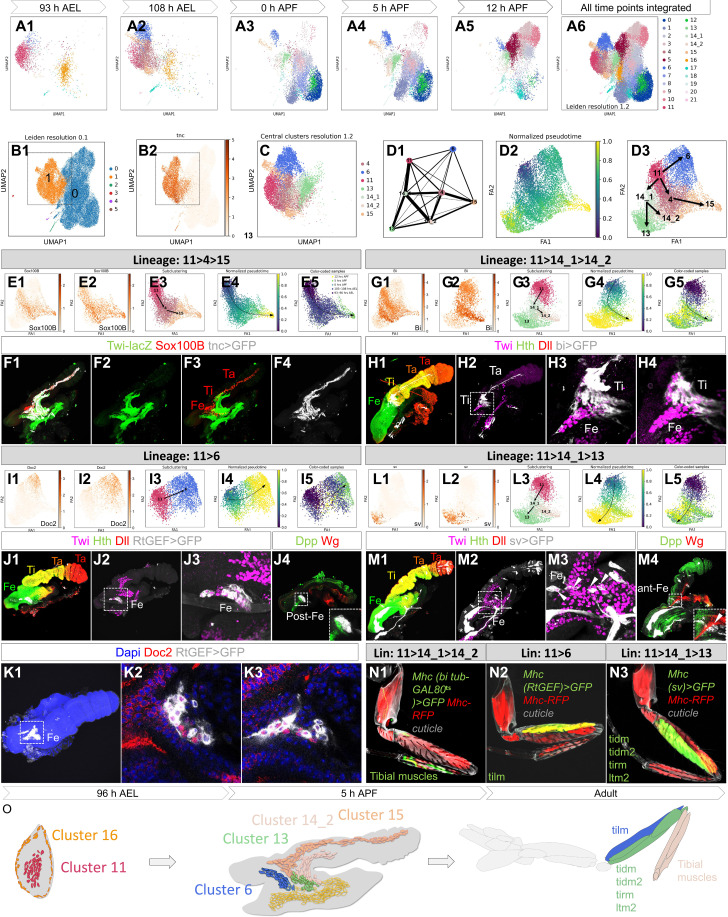
The central cluster of MPs gives rise to four spatially and transcriptionally distinct lineages. (**A1** to **A6**) UMAPs of MPs/myoblasts at different time points [(A1) to (A5)] and a merged UMAP of all time points (A6). (**B1** and **B2**) Low-resolution UMAP revealing two major clusters (B1) and the differential expression of *tnc* (B2) and *unpg* (see fig. S5, A1 to A3). (**C**) High-resolution (1.2) UMAP of the central cluster 1 in (B1). (**D1** to **D3**) PAGA connectivity tree (D1), ForceAtlas2 (FA2) map color coded by pseudotime (D2) or showing distinct central clusters (D3). Arrows indicate the selected lineages based on the PAGA tree, FA2 pseudotime, and real-time FA2 projection [(K5), (G5), (I5), and (L5)]. (**E1** to **M4**) Top: Expression of different lineage markers of the central clusters [(E1), (G1), (I1), and (L1)] and FA2 maps of individual lineages [(E2), (G2), (I2), and (L2)], represented using cluster color code, pseudotime color code, or real-time color code. Bottom: Expression of markers used to determine lineage identity at 5 hours APF: [(F1) to (F4)] Sox100B (red), *twi-lacZ* (green), and *tnc-GAL4>UAS-mCD8::GFP* (gray); [(H1) to (H4)] *bi-GAL4>UAS-mCD8::GFP* (gray), Dll (red), Hth (green), and Twi (purple); [(J1) to (J4) and (K1) to (K3)] *RtGEF-GAL4>UAS-mCD8::GFP* (gray) with Dll (red), Hth (green), and Twi (purple) [(J1) to (J3)]; or with Dpp (green) and Wg (red) (J4) with dashed box highlighting posterior femur (Post-Fe) or with Doc2 (red) and DAPI (blue) [(K1) and (K2)]; (M1) to (M4) *sv-GAL4>UAS-mCD8::GFP* (gray) with Dll (red), Hth (green), Twi (purple) [(M1) to (M3)], or Dpp (green) and Wg (red) (M4) with dashed box highlighting anterior femur (ant-Fe). Arrow indicates myoblasts while asterisks indicate GFP expression in epithelial cells. (**N1** to **N3**) Legs labeled with *Mhc-RFP* (red), showing GFP expression in distal and proximal muscles following lineage tracing from *bi-GAL4* (N1), *RtGEF-GAL4* (N2), or *sv-GAL4* (N3). Note: *tubP-GAL80*^*ts*^ was used to activate lineage tracing after 0 hours APF. (**O**) Schematic summarizing the spatial and temporal lineage tracing of MPs. h/hrs, hours.

To monitor the progressive specification of myoblasts, we performed scRNA-seq at various time points on FACS-sorted GFP^+^ myoblasts (Twi^+^, Mef2^+^; Fig. 5, A1 to A6). After correcting for batch effects, we pooled the data from each time point and generated a low-resolution Uniform Manifold Approximation and Projection (UMAP) (Fig. 5B1). This analysis revealed two major clusters that encompass nearly the entire population of MPs/myoblasts.

To recapture spatial information lost during FACS, we used GAL4 drivers (*tnc-GAL4* and *unpg-GAL4*) that are differentially expressed in the two major clusters 0 and 1 (Fig. 5B2 and fig. S5, A1 to A3). By combining these drivers with morphological (FasIII) and spatial (Dll, Dac, and Hth) markers, we determined that *unpg-GAL4*, which predominantly labels MPs/myoblasts in cluster 0, continues to drive GFP expression at later stages in cells located within the presumptive coxopodite and prothoracic territories (fig. S5, B1 to C2). In contrast, *tnc-GAL4*, which labels all myoblasts in cluster 1, drives GFP expression in cells localized to the telopodite territory (fig. S5, D1 to E2). These observations indicate that, even at late developmental stages, myoblasts remain spatially and transcriptionally subdivided into central and peripheral populations, suggesting the existence of two distinct specification pathways.

We then focused our investigation on the specification of telopodite-associated myoblasts (cluster 1 in Fig. 5B1). To ensure that these populations correspond to myoblasts fated to form telopodite muscles even at later stages, we activated the muscle lineage tracing system during early pupal stage using *tnc-GAL4* in combination with *tub-GAL80^ts^*. This late induction was still sufficient to label all telopodite muscles, confirming *tnc-GAL4* as a reliable marker for telopodite lineages, even at later stages (fig. S6, A1 and A2).

We next generated a higher-resolution UMAP, which revealed six MPs/myoblast clusters (4, 6, 11, 13, 14, and 15; Fig. 5C), and performed a partition-based graph abstraction (PAGA) (*22*) trajectory analysis on these central clusters, identifying four different lineages (Fig. 5, C to D3). This analysis revealed that the central cluster (cluster 11) at 96 hours APF progressively give rise to four muscle lineages (Fig. 5D3; see Materials and Methods). Of note, cluster 14 was subdivided into two subclusters, 14_1 and 14_2, to improve resolution of the lineage trajectories, leading to tibial versus femoral muscles. To recover spatial information lost during FACS and to determine the identity of these lineages, we combined immunostaining, lineage-specific GAL4 drivers, and our muscle lineage tracing system.

Lineage 11 → 4 → 15 continuously expresses *Sox100B* (Fig. 5, E1 to E5). Costaining Sox100B with *tnc-GAL4*-driven GFP, during early pupal stages, revealed tnc^+^/Sox100B^+^ cells in the femur, tibia, and tarsus (Fig. 5, F1 to F4). Their localization in the tarsus, absence of Mef2, and loss of Twist expression at late stages suggest that they are not myoblasts (Fig. 5, F1 to F4; and fig. S6, B1 to B3). Based on their spatial distribution, marker expression confirmed by immunostaining (Lim1^+^ and Sox100B^+^) (Fig. 5, F1 to F4; and fig. S6, B1 to C3), and by comparing marker profiles in our scRNA-seq dataset with previously published data (fig. S6, D1 to E), we propose that these cells correspond to the neural lamella cells recently identified in the adult tarsus (*23*). Our findings extend the presence of this population to the femur and tibia and further demonstrate their mesodermal origin. Consequently, we reclassify the cells previously identified as AMPs (Twi^+^ and Mef2^−^) as mesodermal precursors (MPs), because these cells contribute to more than just muscle formation. We propose to refer to the myoblasts (Twi^+^ and Mef2^+^) that begin to appear at late L3 stages as such (fig. S3A).

The *bi* gene is highly expressed throughout the lineage 11 → 14_1 → 14_2 (Fig. 5, G1 to G5). The *bi-GAL4* enhancer trap line labels Twi^+^ MPs localized in the central region of the leg disc at early stages, corresponding to cluster 11, which gives rise to all telopodite muscles (fig. S4, C1 to C3). However, at 0 and 5 hours APF, *bi-GAL4*–driven GFP^+^/Twi*^+^* myoblasts (cluster 14_2) are restricted to the presumptive tibia region (Fig. 5, H1 to H4; and fig. S6, F1 to F4). In addition, *bi-GAL4* also labels GFP^+^/Twi*^−^* cells located in the presumptive femur, tibia, and tarsus, corresponding to neural lamella cells (cluster 15), which also express *bi* but at lower level (Fig. 5G2 and fig. S6, F1 to F4). To trace the lineage of bi^+^/Twi^+^ myoblasts after pupal stages (cluster 14_2), we activated the muscle lineage tracing system at 0 hours APF using *bi-GAL4* in combination with *tub-GAL80^ts^*. This temporally controlled induction specifically labeled tibial muscles, indicating that myoblasts in cluster 14_2 are committed to the tibial muscle lineage (Fig. 5N1).

To identify lineage 11 → 6, we used *RtGEF-GAL4* to drive GFP expression. We found that this serves as a reliable marker for cluster 6 through development (Fig. 5, J1 to J4; and fig. S6, G1 to G4), as demonstrated by the expression of the transcription factor (TF) Doc2, a specific marker of cluster 6, in RtGEF>GFP^+^ myoblasts (Fig. 5, I1, I2, and K1 to K3; and fig. S6, H1 to H3). These Twi^+^ myoblasts are localized in the presumptive territory of the dorsal femur, where the *tilm* will form (Fig. 5, J1 to K3; and fig. S6, G1 to H3) and lineage tracing of *RtGEF-GAL4*^+^ myoblasts confirmed that they give rise to the *tilm* (Fig. 5N2).

For lineage 11 → 14_1 → 13, we used a *sv-GAL4* line expressed in cluster 13 (Fig. 5, L1 and L2). GFP^+^ myoblasts under the control of *sv-GAL4* were localized in the presumptive territory of the ventral femur only at 5 hours APF (Fig. 5, M1 to M4), which align with their late expression in this lineage as revealed in the PAGA graph (Fig. 5, L1 to L5). Their lineage tracing showed that they give rise to the *ltm2*, *tidm*, *tidm2*, and *tirm* (Fig. 5N3).

**Fig. 6.**
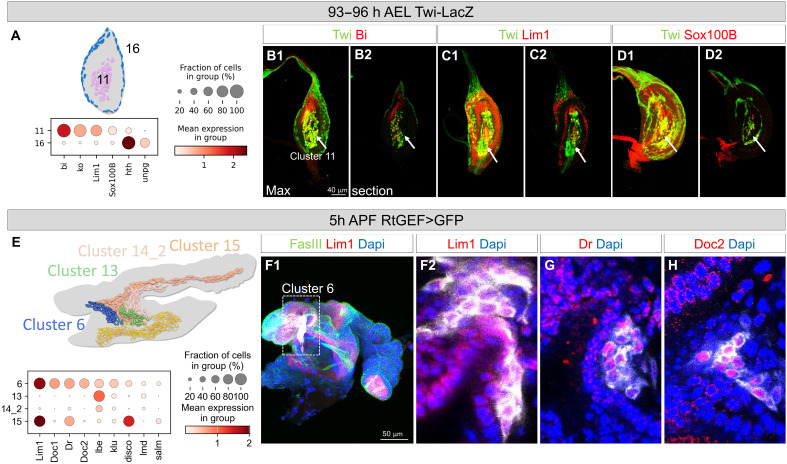
Spatiotemporal dynamics of TF expression. (**A**) Dot plot showing the expression of RNAs coding for TFs differentially expressed in MPs of cluster 11 (central cluster) versus cluster 16 (periphery cluster) at 93 to 96 hours AEL. (**B1** to **D2**) T1 leg discs at 93 to 96 hours AEL immunostained for Twi (green) and Bi (red) [(B1) and (B2)], Lim1 (red) [(C1) and (C2)], or Sox100B (red) [(D1) and (D2)]. [(B1), (C1), and (D1)] Maximum projections; [(B2), (C2), and (D2)] Single confocal sections. Arrows are showing the cluster 11 corresponding to the central cluster. (**E**) Dot plot showing the expression of RNAs coding for TFs differentially expressed in myoblasts of cluster 6 versus the other central clusters (13, 14_2, and 15) at 5 hours APF. (**F1** to **H**) Immature legs at 5 hours APF genetically labeled with *RtGEF-GAL4>UAS-mCD8::GFP* (gray) and immunostained for Lim1 [red, (F1) and (F2)], Dr [red, (G)], or Doc2 [red, (H)]. (F1) is a maximum projection; [(F2) to (H)] are confocal zoom sections showing *RtGEF>GFP^+^* myoblasts in the presumptive femur territory [dashed box in (F1)].

These results reveal that the central populations of MPs, which give rise to distal leg muscles, are progressively specified into subpopulations characterized by distinct combinations of TFs (Fig. 6, A and E). This molecular heterogeneity among them underlies the formation of muscle groups or individual muscles (Fig. 5O). Notably, we show that muscle identity is specified early in development, more than 24 hours before the onset of fusion, for myoblasts in cluster 6, which give rise to the *tilm*. This cluster is already spatially restricted to the future location of the adult muscle and appears uniquely capable of fusing only with cells of the same cluster, suggesting an early, tightly regulated lineage-restricted identity.

### Unique TF codes control muscle diversification

At 93 hours AEL, central and peripheral MPs express distinct combinations of TFs (Fig. 6A), which are likely to play key roles in their respective fate specification. We first confirmed the presence of several TFs at the protein level using available antibodies, validating the expression of peripheral TFs such as Hth (Fig. 4, C3 and C4) and central TFs including Bi, Lim1, and Sox100B (Fig. 6, B1 to D2).

By tracking TF expression in central myoblasts over development, we found that TF codes are dynamically regulated in both space and time. Central TFs such as Bi, Lim1, and Sox100B, which are broadly expressed in all central MPs at 93 hours AEL, become progressively restricted to specific lineages (Fig. 6, A to E). These lineages give rise to three distinct myoblast populations and one population of neural lamella cells, in which additional TFs are activated de novo only at later stages (Fig. 6E). For example, Lim1 is maintained in myoblasts fated to form the *tilm* muscle, whereas Doc1/2 and Dr are specifically induced de novo in these myoblasts at late larval stages. We confirmed the presence of Lim1, Dr, and Doc2 proteins in this lineage by immunostaining (Fig. 6, E to H).

**Fig. 7.**
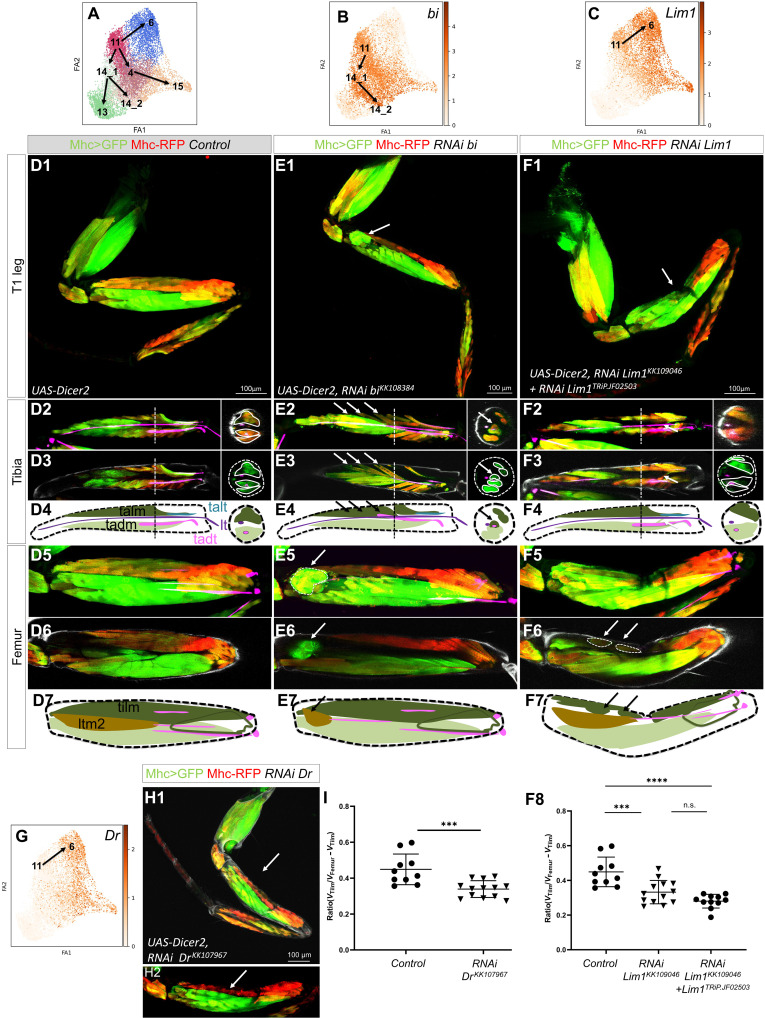
Muscle diversity is controlled by distinct combinations of transcription factors. (**A** to **C**) FA2 map of the central clusters (A) with Bi (B) and Lim1 (C) expression. (**D1** to **F7**) T1 legs genetically labeled with *Mhc-RFP* (red) and *UAS-mCD8::GFP* (green) under the control of *Mhc-GAL4* in a control fly [(D1) to (D7)], a fly with *bi* knockdown (KD) [(E1) to (E7)], and a fly with double *Lim1* KD [(F1) to (F7)]. Note: The two *Mhc* enhancers driving RFP and GAL4 expression have different sequences and display differential muscle activity, allowing us to better distinguish them. [(D2) to (D4), (E2) to (E4), and (F2) to (F4)] Tibia enlargements: max projections [(D2), (E2), and (F2)], confocal sections [(D3), (E3), and (F3)], and schematics [(D4), (E4), and (F4)] showing *talm*, *tadm*, and tendons (*talt*, cyan; *tadt*, pink; *lt*, purple). Cross sections are indicated by dashed lines. In *bi* KD, *talm* is misattached to *tadt* [(E2) to (E4), arrows]. In *Lim1* KD, fibers are occasionally misattached [(F2) to (F4), arrows]. [(D5) to (D7), (E5) to (E7), and (F5) to (F7)] Femur enlargements: max projections [(D5), (E5), and (F5)], confocal sections [(D6), (E6), and (F6)], and schematics [(D7), (E7), and (F7)] showing femoral muscles and tendons (pink; purple labels). In *bi* KD, *ltm2* is rounded and retracted [(E5) to (E7), arrow]. In *Lim1* KD, proximal *tilm* fibers appear detached [(F6) and (F7), arrows]. (**F8**) *tilm* volume quantification in control versus *Lim1* KD showing a muscle volume reduction. ns, not significant, *P* = 0.1491; ***P = 0.0006; ****P < 0.0001. (**G**) FA2 maps showing the expression of *Dr*. (**H1** and **H2**) T1 legs labeled with *Mhc-RFP* (red) and *UAS-mCD8::GFP* (green) under *Mhc-GAL4* in a fly with *Dr* KD in MPs, myoblasts, and muscles, resulting in an atrophic *tilm* (arrow). (**I**) Graph showing the relative volume of the *tilm* in control flies versus *Dr* KD flies in MPs, myoblasts, and muscles. ***P < 0.001.

To assess the functional relevance of Bi and Lim1, two TFs maintained in two distinct lineages (Fig. 7, A to C), we knocked them down using *zfh1-GAL4* and *Mhc-GAL4* to express single or double RNA interference (RNAi) lines that efficiently reduce the expression of both genes (fig. S7, A1 to F3). We chose the *Zfh1-GAL4* driver because it is not expressed during embryogenesis and initiates expression during late larval stages, thereby avoiding early developmental effects and eliminating the need for temporal control of RNAi induction. Notably, only distal muscles (in the femur and tibia) were affected, revealing an instructive role for these TFs in specifying distal muscle identities (Fig. 7, D1 to F7; and figs. S7, A1 to F3, and S8).

When *bi* is knocked down in myoblasts and muscles, some femoral muscles are mildly affected, particularly the *ltm2* (*n* = 9; Fig. 7, E1 and E5 to E7, and table S1), but the strongest phenotypes are observed in tibial muscles (Fig. 7, E1 to E4, and table S1), which normally maintain *bi* expression during pupal stages (Figs. 7B and 5, H1 to H4; and figs. S6, F1 to F4, and S7, A1 and A2). For instance, the tarsus reductor muscle 1 (*tarm1*) is occasionally absent (*n* = 1) or reduced in fiber number (*n* = 8; fig. S8, D1 to D4), whereas the tarsus levator muscle (*talm*) muscle is consistently affected (*n* = 14; Fig. 7, E1 to E4; fig. S8, D5 to D7; and table S1). In some cases, *talm* fibers appear rounded and fail to attach to their correct distal tendon in adults (fig. S8, D5 to D7, and table S1). However, the most consistent phenotype involves *talm* fibers misattaching to the tarsus depressor muscle (*tadm*) tendon (*tadt*), pulling this tendon toward the center of the tibia near the long tendon (*lt*) (Fig. 7, E2 to E4).

Upon *Lim1* knockdown using a combination of two different RNAi lines, tibial muscles are generally weakly affected. In some cases, small fibers target incorrect tendons (Fig. 7, F1 to F4), but the most frequent phenotype in the tibia is an increased number of *tarm1* fibers (*n* = 4 of 11), occasionally resulting in a complete duplication of this muscle (fig. S8, C1 to C4). In contrast, several phenotypes show a folded femur at the level of the *tilm* (*n* = 6 of 11). In these folded legs, we often observe rounded *tilm* fibers that fail to attach to tendons (Fig. 7, F1 to F7). The remaining *tilm* fibers are atrophic, showing a strong reduction in volume that correlates with the number of RNAi lines used to knock the gene (Fig. 7F8).

Notably, when *Dr*, a gene whose expression is induced at late larval stages (Fig. 7G and fig. S7, G1 to G3), is knocked down in myoblasts and muscles, only the *tilm* muscle is affected, becoming severely atrophic (Fig. 7, H and I; and fig. S7, H1 to H3). Most legs analyzed under these conditions are folded.

Together, these results demonstrate that central myoblasts rely on dynamic and spatially restricted combinations of TFs to define specific distal muscle identities. Individual TFs within these codes—such as Bi, Lim1, and Dr—play highly specific and instructive roles, revealing a modular regulatory logic underlying muscle diversification in the developing leg.

### Wg and Dpp secreted by the epithelium drive the first step of MP specification

Our data reveal that MPs progressively transition from a naïve state through a finely tuned, multistep process that ultimately specifies MPs to generate muscle diversity. The first step of specification distinguishes MPs located at the center of the disc from those at the periphery, which give rise to distal and proximal muscles, respectively.

**Fig. 8.**
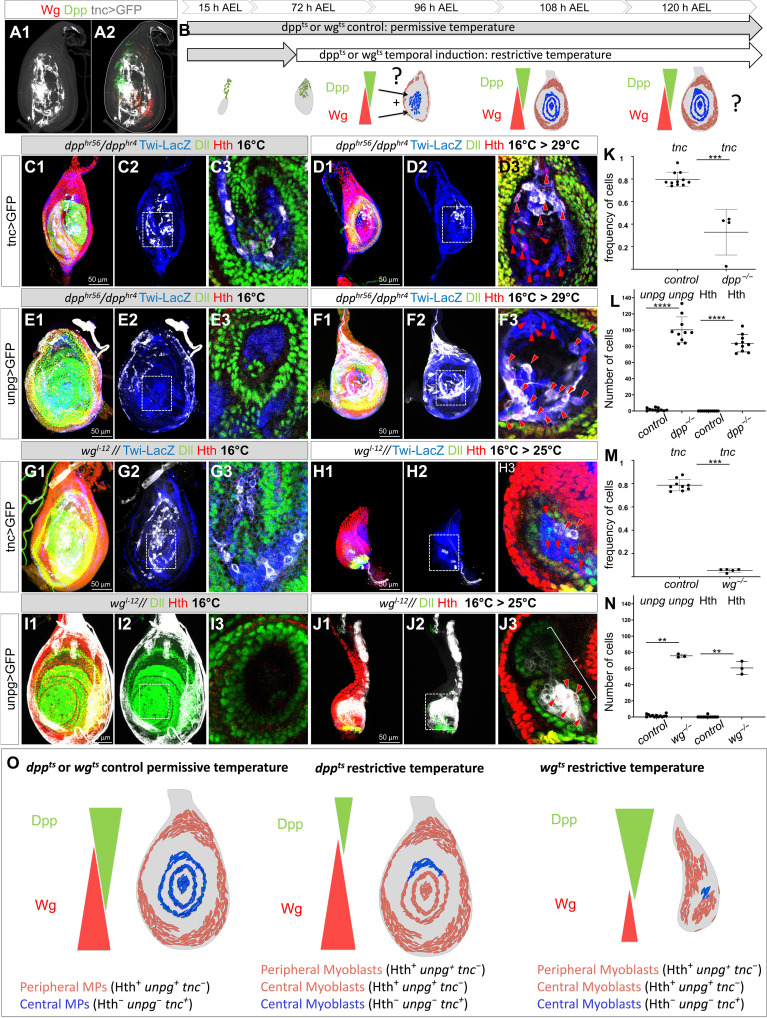
Wg and Dpp secreted by the epithelium act synergistically to specify the central versus peripheral identity of MPs. (**A1** and **A2**) Late third instar T1 leg disc labeled with *tnc-GAL4>UAS-mCD8::GFP* (gray), immunostained for Dpp (green) and Wg (red). (**B**) Schematic of the conditional *dpp* and *wg* loss-of-function strategy. (**C1** to **F3**) Third instar leg discs of *twi-lacZ*; *dpp^hr56^/dpp^hr4^* mutants expressing *tnc-GAL4>UAS-mCD8::GFP* (gray) [(C1) to (D3)] or *unpg-GAL4>UAS-mCD8::GFP* (gray) [(E1) to (F3)], immunostained for Dll (green), Hth (red), and Twi (blue). Discs were kept at the permissive temperature [(C1) to (C3) and (E1) to (E3)] or shifted to the nonpermissive temperature shortly before the specification of central versus peripheral MPs [(D1) to (D3) and (F1) to (F3)]. Red arrowheads indicate Hth^+^ myoblasts in the center of the disc. Outlined red arrowheads indicate Hth^+^ myoblasts that also express *tnc>GFP* or *unpg>GFP*. (**G1** to **J3**) Third instar leg discs of *twi-lacZ;* // mutants expressing *tnc>GFP* [(G1) to (H3)] or *unpg>GFP* [(I1) to (J3)]. Discs were stained for Dll (green), Hth (red), and Twi (blue) [(G1) to (H3)] or for Dll and Hth only [(I1) to (J3)]. Larvae were kept at permissive temperature [(G1) to (G3) and (I1) to (I3)] or shifted to nonpermissive temperature before central/peripheral MP specification [(H1) to (H3) and (J1) to (J3)]. Red arrowheads mark central Hth^+^ myoblasts; outlined arrowheads mark Hth^+^ cells expressing *tnc>GFP* or *unpg>GFP*. Bracket indicates all *unpg>GFP* myoblasts. See fig. S10, showing that the *unpg>GFP* and Hth^+^ cells in (J1) to (J3) are Twi^+^. (**K** to **N**) Quantification of tnc^+^ [(K) and (M)], unpg^+^, and Hth^+^ [(L) and (N)] myoblasts in *wg*^1-12^// or *dpp*^hr56^/*dpp*^hr4^ mutant flies raised at the permissive (control) or restrictive temperature (*wg*^−^/^−^ or *dpp*^−^/^−^). [(K) and (M)] Relative proportion of tnc^+^ myoblasts was calculated rather than the absolute number of cells, because the total number of myoblasts is reduced in *wg*^1-12^// mutants raised at the restrictive temperature (small discs). (**O**) Schematic of the phenotypic outcomes. h, hours. (K) ***P < 0.0007; (L) ****P < 0.0001; (M) ***P < 0.0005; (N) **P <0.0018.

Wg (Wnt1) and Dpp [bone morphogenetic protein (BMP)] are two morphogens secreted by the ventral and dorsal epithelium, respectively (*24*). We found that MPs at the center of the disc specified to produce telopodites muscles are localized directly beneath the sources of both morphogens (Fig. 8, A1 and A2). Although myoblasts do not secrete these morphogens, they express the necessary receptors to respond to them (fig. S9).

To test the role of Wg and Dpp in this process, we used temperature-sensitive alleles of *wg* and *dpp* to inactivate their functions before the initial step of MP specification (proximal versus distal fate specification). When either Wg or Dpp signaling was disrupted, we observed a partial transformation of central myoblasts (tnc^+^, unpg^−^, and Hth^−^), which adopted a peripheral fate (tnc^−^, unpg^+^, and Hth^+^) (Fig. 8, C1 to N). While a large fraction of central myoblasts was fully transformed in the absence of Wg or Dpp, others displayed intermediate phenotypes, coexpressing markers of both identities (tnc^+^, Hth^**+**^ or tnc^−^, Hth^−^; unpg^−^, Hth^+^ or unpg^+^, Hth^−^) (Fig. 8, D3, F3, H3, J3, and K to N; and fig. S10).

In the absence of Wg, leg discs were smaller, likely due to impaired cell proliferation, a well-established function of Wg (*25*, *26*), as well as a reduction in the telopodite territory within the epithelium, reflecting Wg’s role in patterning. Nevertheless, the epithelium continued to express Dll, and the central cluster of myoblasts remained positioned beneath Dll^+^ epithelial cells, allowing us to identify these cells as telopodite myoblasts that had been converted to a peripheral identity (Fig. 8, G1 to J3). Under these conditions, the number of myoblasts was also markedly reduced (Fig. 8, M and N), a phenotype that was also observed, albeit to a lesser extent, when flies carrying the temperature-sensitive *dpp* allele were raised at the nonpermissive temperature (Fig. 8, C1 to F3, K, and L).

These findings indicate that the synergistic action of Wg and Dpp is essential for the initial specification of myoblasts into proximal versus distal fate, which appear to represent the ground state (Fig. 8O).

### Wg and Dpp directly control the first step of MP specification

**Fig. 9.**
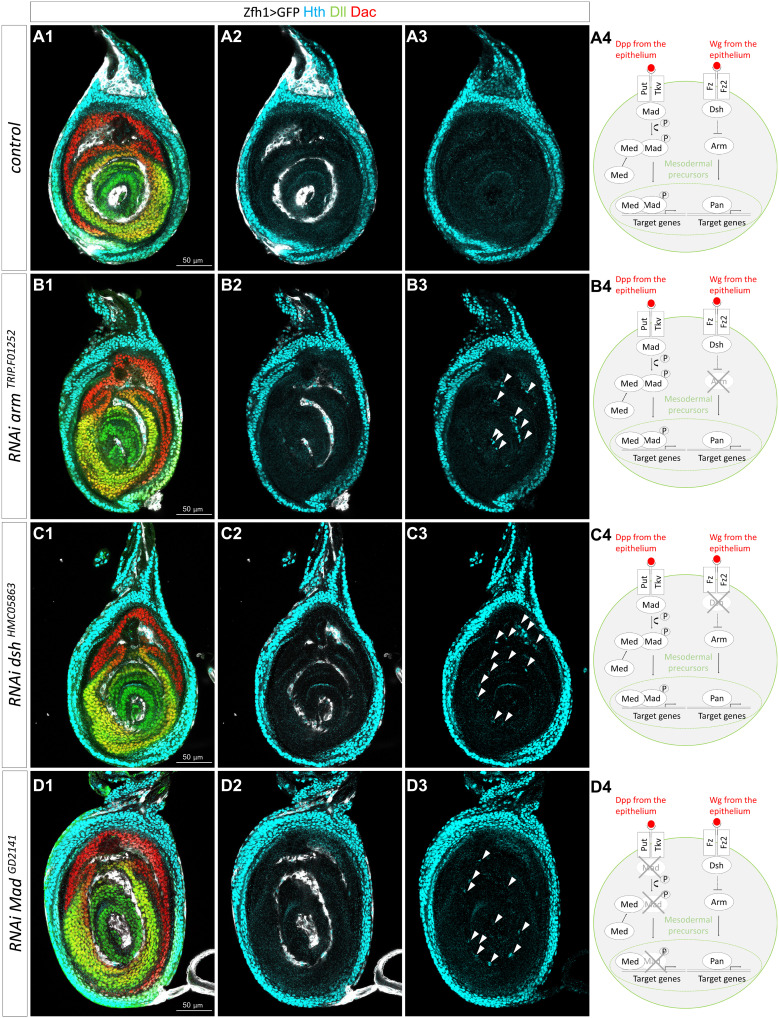
Wg and Dpp signaling is required cell-autonomously in MPs to specify the central versus peripheral MPs identities. (**A1** to **D4**) Late third instar T1 leg disc genetically labeled with *zfh1-GAL4>UAS-mCD8::GFP* (gray) and immunostained for Dll (green), Dac (red), and Hth (cyan) in control flies [(A1) to (A3)] or following RNAi-mediated knockdown of *arm* [(B1) to (B3)], *dsh* [(C1) to (C3)], and *Mad* [(D1) to (D3)] flies. White arrowheads indicate Hth^+^ MPs in the center of the discs. [(A4), (B4), (C4), and (D4)] Schematics of the Dpp and Wg signaling pathways in MPs. Crosses indicated the different RNAi knockdown conditions.

To determine whether Wg and Dpp act directly on MPs, we specifically knocked down key components of the Wg and Dpp signal transduction pathways (Fig. 9, A4, B4, C4, and D4) in MPs using *zfh1-GAL4*, a driver that is not expressed in epithelial cells (fig. S7).

As expected, knockdown of *arm* or *dsh* (Wg pathway), or *Mad* (Dpp pathway), in MPs did not affect epithelial patterning (Fig. 9, A1, B1, C1, and D1), in contrast to *wg* or *dpp* mutant discs (Fig. 8). However, under these conditions, central MPs, which are normally Hth negative, began to express Hth, demonstrating that Wg and Dpp signaling is required cell-autonomously in MPs to maintain their central identity and prevent adoption of a peripheral fate (Fig. 9, A2, A3, B2, B3, C2, C3, and D1 to D3).

### Wg and Dpp also control the second step of myoblast specification

Following the initial segregation into proximal and distal populations, myoblasts undergo further specification into distinct muscles. To investigate the developmental logic underlying muscle diversity, we focused on the specification of lineage 11 → 6, which gives rise to the *tilm* muscle (Fig. 5).

**Fig. 10.**
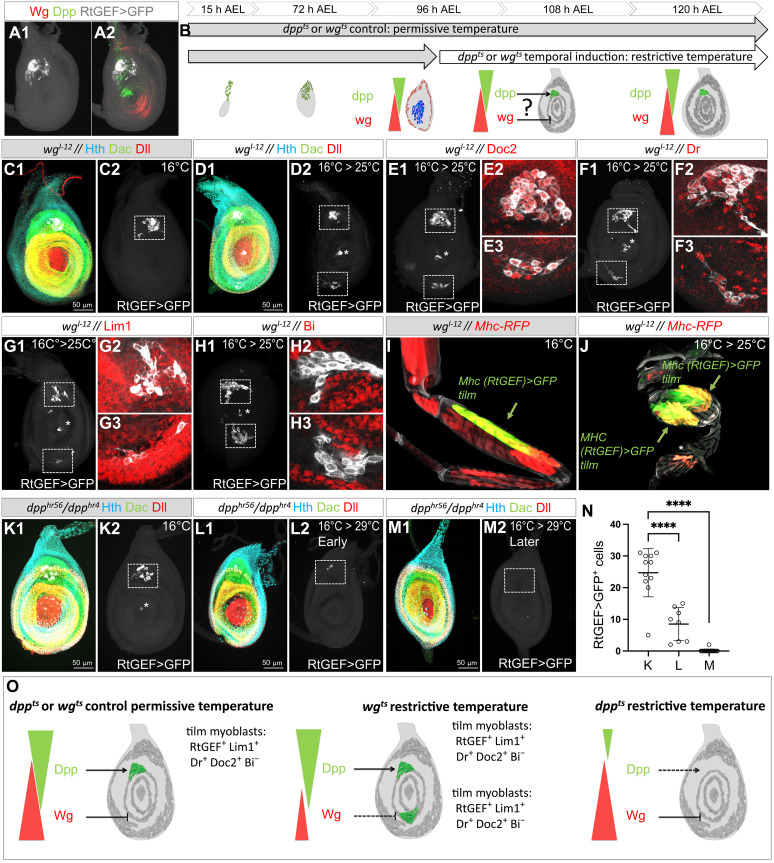
Wg and Dpp act antagonistically to specify MPs to give rise to the *tilm*, dorsal muscle. (**A1** and **A2**) Late third instar T1 leg disc labeled with *RtGEF-GAL4>UAS-mCD8::GFP* (gray), immunostained for Dpp (green) and Wg (red). (**B**) Schematic of the conditional *dpp* and *wg* loss-of-function strategy. (**C1** to **H3**) Third instar leg discs of *wg^1-12^*// mutants expressing *RtGEF-GAL4>UAS-mCD8::GFP* (gray). Discs were immunostained for Dac (green), Dll (red), and Hth (cyan) [(C1) to (D2)] or Doc2 (red) [(E1) to (E3)] or Dr (red) [(F1) to (F3)] or Lim1 (red) [(G1) to (G3)] or Bi [(H1) to (H3)]. Samples were maintained at the permissive temperature [(C1) to (C2)] or shifted to the nonpermissive temperature after the specification of central versus peripheral MPs [(D1) to (H3)]. Asterisks show *RtGEF-GAL4>UAS-mCD8::GFP* myoblast occasionally seen in the center of the discs (dashed box and zoom panels). (**I** and **J**) Adult T1 leg disc of *wg^1-12^*// mutants labeled with *Mhc-RFP* (red), showing GFP expression (green) in the *tilm* muscle (green arrow) following lineage tracing from *RtGEF-GAL4.* Samples were maintained at the permissive temperature (I) or shifted to the nonpermissive temperature after the specification of central versus peripheral MPs (J). We also detected a GFP^+^ tibial muscle, occasionally duplicated, when flies were maintained at the nonpermissive temperature [asterisk in (J)]. (**K1** to **M2**) Third instar leg discs of *dpp^hr56^/dpp^hr4^* mutants expressing RtGEF>GFP. Discs were stained for Dac (green), Dll (red), and Hth (cyan). Larvae were kept at permissive temperature [(K1) and (K2)] or shifted to nonpermissive temperature after central/peripheral MP specification at late [(L1) and (L2)] or early [(M1) and (M2)] stages. Asterisks mark occasional RtGEF^+^ cells in the disc center. (**N**) Graph quantifying GFP^+^ myoblasts in *dpp^hr56^/dpp^hr4^* mutants at permissive temperature (K) or after temperature shift at early (L) or late (M) stages after central/peripheral MP specification. ****P < 0.0001. (**O**) Schematic summary of the phenotypic outcomes observed in (C1) to (M2). h, hours.

Myoblasts in cluster 6 express a specific combination of TFs (Fig. 6E) and localize near the Dpp source (Fig. 10, A1 and A2), suggesting that Dpp promotes the specification of *tilm* myoblasts. Conversely, Wg may repress their specification in the ventral region (Fig. 10B).

To test this hypothesis, we inactivated Wg or Dpp signaling after the initial peripheral/central specification step (96 hours AEL). In *wg* mutants, we consistently observed a de novo cluster of RtGEF>GFP^+^ myoblasts in the ventral region of the disc in all sample analyzed (Fig. 10, C1 to D2 and O). Costaining with antibodies against TFs expressed in *tilm* myoblasts or other myoblast populations revealed that this ectopic cluster represents a duplication of the *tilm* myoblast identity (Doc^+^, Dr^+^, Lim^+^, and Bi^−^) in the presumptive ventral domain (Fig. 10, E1 to H3). Moreover, to determine whether this duplication leads to the formation of an additional *tilm* muscle, we lineage-traced both clusters through to the adult stage and observed the presence of a duplicated *tilm* muscle in adults (Fig. 10, I and J).

Conversely, in *dpp* mutants, central MPs failed to adopt the *tilm* fate (cluster 6), as revealed by a strong reduction or absence of RtGEF>GFP^+^ cells depending on Dpp inactivation timing (Fig. 10, K1 to O). These results indicate that the number of myoblasts specified to form the *tilm* muscle depends on the duration of their exposure to Dpp signaling.

These findings demonstrate that, beyond their role in early myoblast segregation, Wg and Dpp signaling continue to control the second step of myoblast specification by spatially restricting the identity and positioning of *tilm* precursors, thereby ensuring the correct patterning of adult leg muscles (Fig. 10O).

## DISCUSSION

### Group of myoblasts are specified before fusion to generate muscle diversity

How individual muscle identities are specified during development remains a central question in developmental biology. We demonstrate that mesodermal precursors (MPs) undergo stepwise transitions from a naïve state to acquire distinct identities, thereby specifying muscle diversity before fusion. This mechanism shows that MPs and myoblasts are not passive actors simply following the prepattern established by connective tissue cells such as tendons. Instead, disrupting the expression of transcription factors such as Bi in specific muscle lineages leads to incorrect tendon connectivity, revealing that communication between the two tissues is both specific and, at least in part, intrinsically encoded within muscle lineages.

Lineage tracing revealed that MPs remain multipotent until mid-larval stages, after which they become progressively restricted through a temporally and spatially regulated program. The first major bifurcation separates central and peripheral MP populations within the leg imaginal disc, which subsequently give rise to distal and proximal muscles, respectively. These territories are defined by distinct TF expression profiles: Lim1, Bi, Ko, and Sox100B in the central region, and Hth and unpg in the peripheral domain.

As development proceeds, these broad populations further diversify into transcriptionally distinct subpopulations, each prefiguring the position of specific muscle groups or individual muscles. Intriguingly, we also identified a lineage of mesodermal cells in the distal leg that gives rise to neural lamella cells, suggesting broader developmental potential than previously appreciated. Accordingly, we propose replacing the term AMPs with MPs to better reflect this expanded identity.

We stopped our analysis at early pupal stages because we identified a lineage already committed to forming a single muscle at this stage, validating our model, and demonstrating how specific transcriptional signatures emerge early. Although our current dataset captures early diversification, we expect that later time points would reveal additional fate bifurcations, leading to full muscle patterning.

### The driving force of MP/myoblast specification is provided by morphogens secreted from the epithelium

Our data demonstrate that morphogens secreted by the epithelium are key drivers of myoblast specification, enabling the spatiotemporal coordination of epithelial and mesenchymal development. By following the developmental trajectory of the *tilm* (a distal leg muscle), we found that morphogens function in multiple, temporally distinct phases. Initially, Wg and Dpp act synergistically to specify central MPs toward a distal muscle fate; in the absence of either signal, MPs adopt a proximal identity. At later stages, Dpp promotes specification of dorsal central myoblasts into the *tilm* lineage, while Wg represses this fate in the ventral region.

### Diversity of cellular and syncytial fates

Most biological systems, such as the vascular or nervous systems, achieve their function through the generation of cells with distinct identities. These identities are built during hematopoiesis or neurogenesis by mechanisms that generate cellular diversity in space and time (*27*–*31*). A well-characterized example is the generation of neural diversity in *Drosophila*, where spatial and temporal selectors (RNA binding proteins or transcription factors) define the identity of neural stem cells, leading to the production of postmitotic neurons that express terminal selectors determining their final fate (*27*–*30*, *32*, *33*). In contrast, the locomotor system achieves part of its function through the generation of distinct muscles, which are syncytial organs formed by cell fusion. Consequently, the developmental logic is different: Groups of myoblasts undergo a shared spatiotemporal program of transcription factor deployment before fusion, ensuring that cells destined to build the same muscle synchronize their transcriptional states. This coordination is mediated by morphogen signals secreted from the epithelium. We hypothesize that this mechanism ensures that the resulting syncytium inherits a coordinated transcriptional identity, which is essential for building a unique muscle.

### Evolution of leg appendages in arthropods

In an evolutionary context, our study supports the classical division of the arthropod limb into coxopodite and telopodite (*34*). The prevailing theory suggests that the telopodite, the “true leg,” evolved from the coxopodite, which is considered to be part of the thoracic body wall. In *Drosophila*, this theory is supported by numerous studies showing that the molecular signature of the epithelial cells of the coxopodite (comprising the coxa and trochanter) closely resembles that of the trunk epithelium, whereas the epithelial cells of the telopodite display a distinct molecular identity.

In the *Drosophila* leg imaginal disc, the central region exposed to both major morphogens, wg and Dpp, gives rise to the telopodite (Dll^+^ and Dac^+^), whereas the peripheral region differentiates into the prothorax and coxopodite (Hth^+^). This early organization of the leg epithelium is paralleled by a patterning in the mesoderm. Peripheral MPs that generate the coxa and trochanter muscles (coxopodite) share transcriptomic signatures with prothoracic MPs, whereas telopodite MPs (femur, tibia, and tarsus) form a transcriptionally distinct population. This supports the view that the telopodite constitutes a true appendage, whereas the coxopodite represents an extension of the body wall.

This mechanism allows the coordinated development of the epithelium and the mesodermal derivatives, the MPs, while also providing evolutionary plasticity. Because the epithelium is the source of morphogens, changes in its patterning will also reshape the patterning of mesodermal derivatives.

### A potential mechanism conserved in vertebrates

In vertebrate limb development, MPCs migrate from the somites into the limb bud, where local signals, largely derived from a prepattern established by connective tissue cells, determine their final positioning (*8*–*11*). In this classical model, muscle precursors are viewed as developmentally “naïve,” passively following the prepattern established by the connective tissues.

However, several studies challenge this view and instead support a model of early lineage restriction. For instance, MPCs at early developmental stages exhibit bipotency, retaining the ability to differentiate into both muscles and endothelial cells in response to environmental signals (*35*). This plasticity mirrors what we observed in *Drosophila*, where MPs can also give rise to nonmuscle cell types, such as neural lamella cells.

A further layer of evidence suggests that muscle diversification may not result solely from the passive positioning of myoblasts. Adult muscles also display heterogeneity in their sarcomeric organization. For instance, myosin heavy chain (MHC) proteins, the main components of sarcomeres, exist in several isoforms that are differentially expressed across muscle groups under the control of a muscle-specific super enhancer. This regulation suggests that muscles follow distinct developmental transcriptional programs rather than converging on a single default pathway (*36*).

Additional evidence for an active specification into specific muscles comes from the spatiotemporal expression patterns of HOXA11 and HOXA13 in vertebrate limb muscle precursors, which suggest that myoblasts are already prespecified to contribute to particular muscle lineages. Notably, BMP signaling from epithelial cells has been implicated in regulating Hox gene expression in these cells, reinforcing the idea that positional signals influence MPC identity from early developmental stages (*37*–*40*). In line with this, although not explored in depth here, we observed differential Hox gene expression in early myoblasts of the *Drosophila* leg disc, with Antp expressed in central myoblasts and Scr in peripheral ones. These findings suggest that the spatiotemporal control of MP identities by epithelial cells may represent a conserved mechanism guiding muscle lineage diversification across species.

This logic of early lineage restriction may also have implications for human disease. Several muscle-specific myopathies, including muscular dystrophies that selectively affect certain muscle groups while sparing others (*41*, *42*), may reflect an underlying developmental lineage–based organization of muscles. Such patterns highlight that muscles are not functionally or molecularly homogeneous but, instead, may retain distinct embryonic origins and regulatory programs that influence their disease susceptibility.

## MATERIALS AND METHODS

### Experimental model and subject details

The experimental model for this study was the vinegar fly *Drosophila melanogaster*. A complete list of strains used in this study is provided in the Key Resources Table (Table 1) and in the Genetic Crosses (Table 2) section for each figure. Unless otherwise specified, flies were maintained on standard cornmeal medium at 25°C under a 12:12-hour light-dark cycle. Unless stated otherwise, males and females were chosen at random.

**Table 1.** Key resources. DSHB, Developmental Studies Hybridoma Bank; BDSC, Bloomington Drosophila Stock Center.

Reagent or resource	Source	Identifier
Antibodies		
Donkey anti-chicken Alexa 488	Jackson ImmunoResearch	Catalog no. 703-145-155; RRID: AB_2340375
Donkey anti-guinea pig DyLight 405	Jackson ImmunoResearch	Catalog no. 706-475-148; RRID: AB_2340470
Donkey anti-mouse Cy3	Jackson ImmunoResearch	Catalog no. 715-165-151; RRID: AB_2315777
Donkey anti-rat Alexa 647	Jackson ImmunoResearch	Catalog no. 712-605-153; RRID: AB_2340694
Goat anti-chicken Alexa 647	Abcam	Catalog no. ab150171
Goat anti-mouse Alexa 405	Abcam	Catalog no. ab175661
Goat anti-rat Alexa 555	Abcam	Catalog no. ab150166
Goat anti-rabbit Alexa 405	Abcam	Catalog no. ab175653
Goat anti-mouse Alexa 647	Invitrogen	Catalog no. A21236; RRID: AB_2535805
Goat anti-rabbit Alexa Plus 555	Invitrogen	Catalog no. A32732; RRID:AB_2633281
Goat anti-rabbit Alexa Plus 647	Invitrogen	Catalog no. A32733; RRID:AB_2633282
Phalloïdin Alexa 546	Invitrogen	Catalog no. A22283
Mouse anti–β-galactosidase	Promega	Catalog no. Z3781
Chicken anti–β-galactosidase	Abcam	Catalog no. ab134435
Mouse anti-Dachshund	DSHB	Catalog no. ab579773 (mAbdac1-1-s)
Mouse anti–Fasciclin III	DSHB	Catalog no. ab528238 (7G10 anti–Fasciclin III)
Mouse anti-Wg	DSHB	Catalog no. ab528512 (4D4)
Rabbit anti-Bi(omb)	Gift from S. Matsuda	
Rabbit anti-Doc2	Gift from I. Reim	
Rabbit anti-Dpp	Gift from A. Takuya	
Rabbit anti-Hth	Gift from R. S. Mann	
Rabbit anti-Lim1	Gift from R.S. Mann	
Rabbit anti-Mef2	Gift from K. Domsch	
Rabbit anti-Msh (Dr)	Gift from C. Doe	
Rabbit anti-Sox100B	Gift from X. Rongwen	
Rabbit anti-Tenectin	Gift from S. Fraichard	
Rabbit anti-Twist	Gift from Y. Ghavi-Helm	
Guinea pig anti-Dll	Gift from R.S. Mann	
Chemicals, peptides, and recombinant proteins		
Formaldehyde	Thermo Fisher Scientific	Catalog no. 28908
PBS	Dutscher	Catalog no. X0515-500
Absolute ethanol	Cristalco	Catalog no. 64-17-5
Vectashield antifade mounting medium	Vector Laboratories	Catalog no. H1000
Vectashield antifade mounting medium with DAPI	Vector Laboratories	Catalog no. H1200
Triton X-100	Sigma-Aldrich	Catalog no. T8787-100mL
Bovine serum albumin	Sigma-Aldrich	Catalog no. A7906-500g
Sodium hypochlorite solution (6 to 14% active chlorine)	Sigma-Aldrich	Catalog no. CAS 7681-52-9
Heptane	Sigma-Aldrich	Catalog no. 32287-1L-M CAS 142-82-5
Shields and Sang M3 Insect Medium	Sigma-Aldrich	Catalog no. S3652-500ML
Collagenase type 1	Sigma-Aldrich	Catalog no. C2674-100MG
Papain	Sigma-Aldrich	Catalog no. P4762-100MG
Fetal bovine serum	Sigma-Aldrich	Catalog no. F2442-100ML
Critical commercial assays		
Chromium Single Cell 3′ Reagent Kit version v3.1	10x Genomics	
Experimental models: *D. melanogaster*/strains		
Mhc-RFP (second chromosome)	BDSC	RRID:BDSC_38464
UAS>GFP: P{w[+mC]=UAS-mCD8::GFP.L}LL5	BDSC	RRID:BDSC_60707
UAS>GFP: P{w[+mC]=UAS-mCD8::GFP.L}LL6	BDSC	RRID:BDSC_60726
UAS-FB1.1: P{y[+t*] w[+mC]=UAS-Flybow.1.1}VIE-260B	BDSC	RRID:BDSC_35537
tnc-Gal4: PBac{w[+mC]=IT.GAL4}2090-G4	BDSC	RRID:BDSC_65718
bi-Gal4: y[1] w[*] P{w[+m*]=GAL4}MD735	BDSC	RRID:BDSC_80153
ko-Gal4: Mi{Trojan-GAL4.0}ko[MI10038-TG4.0]	BDSC	RRID:BDSC_76207
unpg-Gal4: TI{GFP[3xP3.cLa]=CRIMIC.TG4.1}unpg[CR00787-TG4.1]	BDSC	RRID:BDSC_83193
UAS-FLP : P{w[+mC]=UAS-FLP.D}JD1	BDSC	RRID: BDSC_4539
Tub-GAL80[ts] : P{w[+mC]=tubP-GAL80[ts]}	BDSC	RRID: BDSC_7016
sv-Gal4: TI{GFP[3xP3.cLa]=CRIMIC.TG4.2}sv[CR00370-TG4.2]	BDSC	RRID:BDSC_78901
RtGEF-Gal4: PBac{w[+mC]=IT.GAL4}RtGEF[4046-G4]	BDSC	RRID:BDSC_77656
UAS-Dcr2: P{w[+mC]=UAS-Dcr-2.D}10	BDSC	RRID:BDSC_24651
Lim1-RNAi: P{y[+t7.7] v[+t1.8]=TRiP.JF02503}attP2	BDSC	RRID:BDSC_29341
Dll-GAL4 : P{w[+mW.hs]=GawB}Dll[md23]	BDSC	RRID:BDSC_3038
24B-GAL4 : P{w[+mW.hs]=GawB}how[24B]	BDSC	RRID:BDSC_1767
Mef2-GAL4 : P{w[+mC]=GAL4-Mef2.R}3	BDSC	RRID:BDSC_27390
dpp[hr56]	BDSC	RRID:BDSC_36528
dpp[hr4]	BDSC	RRID:BDSC_81062
wg[l-12]	BDSC	RRID:BDSC_7000
P{w[+mC]=Tub-GAL4DBD.D}2	BDSC	RRID:BDSC_60298
Oregon-R-C	BDSC	RRID:BDSC_5
bi-RNAi: P{KK108384}VIE-260B	VDRC	RRID:VDRC_v100598
Lim1-RNAi: P{KK109046}VIE-260B	VDRC	RRID:VDRC_v104468
Msh/Dr RNAi: P{KK107967}VIE-260B	VDRC	RRID:VDRC_v110625
Arm-RNAi: y[1] v[1]; P{y[+t7.7] v[+t1.8]=TRiP.JF01252}attP2	BDSC	RRID:BDSC_31305
Dsh-RNAi: y[1] sc[*] v[1] sev[21]; P{y[+t7.7] v[+t1.8]=TRiP.HMC05863}attP40	BDSC	RRID:BDSC_64989
Mad-RNAi: w1118; P{GD4121}v12635	VDRC	RRID:VDRC_v12635
hs-mFLP5	Flybase	FBti0161261
zfh1-T2A-GAL4	Flybase	FBal0340857
Mhc-GAL4	This study	N/A
Mhc>>GAL4 (second chromosome)	This study	N/A
Mhc>>GAL4 (third chromosome)	This study	N/A
twi-LacZ (X chromosome)	Gift from K. Millen	N/A
twi-LacZ (second chromosome)	Gift from K. Millen	N/A
Lim1-VP16: w1118; Lim1-T2A-VP16/FM7;+;+	Gift from C. Desplan	N/A
Software and algorithms		
Amira 3D software (version 6.2)	SCR_007353	www.fei.com/
ImageJ (version 2.16)	Schneider *et al.* (*57*)	https://imagej.nih.gov/ij/
R (version 4.2.3)	R Foundation for Statistical Computing	www.r-project.org/
Python (version 3.10.11)	Python Software Foundation	www.python.org/
Prism 10 (version 10.4.0)	GraphPad Software	www.graphpad.com/features
Cell Ranger (version 5.0.0)	Zheng *et al.* (*45*)	www.10xgenomics.com/support/software/cell-ranger/downloads/previous-versions
Seurat (version 4.1.1)	Hao *et al.* (*58*)	https://satijalab.org/seurat/
Scanpy (version 1.9.1)	Wolf *et al.* (*48*)	https://scanpy.readthedocs.io/en/stable/
Imaris (version 10.2)	Oxford Instruments	https://imaris.oxinst.com/
Others		
BD FACSAria II		
TapeStation 2200		
Illumina NextSeq 500 sequencer		
10x Genomics Chromium Controller		

**Table 2.** Genetic crosses.

Figures	Summary	Genetic crosses
Fig. 1	Mhc labeling	*y, w; Mhc-GAL4(attP40)/CyO; TM6b/MKRS*
		TO *yw, hs-FLP1.22; UAS-mCD8::GFP/CyO; TM6b/MKRS*
Fig. 2A	Dll labeling	Stock *y, w; Dll-GAL4, UAS-mCD8::GFP/CyO; UAS-mCD8::GFP/MKRS*
Fig. 2 (B and C)	24B labeling	*y, w, hs-FLP1.22; If/CyO; 24B-GAL4/TM6b*
		TO *y, w,hs-FLP1.22; UAS-mCD8::GFP/CyO; TM6b/MKRS*
Fig. 2D	Twi labeling	Stock *w; twi-LacZ//; +*
Fig. 2 (E to H)	Mef2 labeling	Stock *y, w; UAS-mCD8::GFP, Mhc-RFP/CyO; Mef2-GAL4/TM6b*
Fig. 3	Flybow	*y, w; UAS-FB1.1/CyO; Mef2-GAL4/TM6b*
		TO *hs-mFLP5//;+;+*
Figs. 4 (A and B), 5, and 6	scRNA-seq 93–96 hours AEL + 108–111 hours AEL	*twi-GAl4//; 24B-GAl4//*
		TO *y,w, hs-Flp1.22; UAS-mCD8::GFP; MKRS/TM6b*
Fig. 4C	unpg and twi labeling	*w; TI{GFP[3xP3.cLa]=CRIMIC.TG4.1}unpg[CR00787-TG4.1], UAS-mCD8::GFP, Mhc-RFP/CyO; MKRS/TM6b*
		TO *twi-LacZ//;+;+*
Fig. 4D	tnc and twi labeling	*y, w, hs-FLP1.22; UAS-mCD8::GFP, Mhc-RFP/CyO; PBac{w[+mC]=IT.GAL4}2090-G4/TM6b*
		TO *y, w; twi-LacZ/CyO;+*
Fig. 4E	tnc labeling	Stock *y, w, hs-FLP1.22; UAS-mCD8::GFP, Mhc-RFP/CyO; PBac{w[+mC]=IT.GAL4}2090-G4/TM6b*
Fig. 4F	tnc memory system	*y, w; Mhc>>GAL4 (attP40), Mhc-RFP, UAS-mCD8::GFP/CyO; Mhc>>GAL4 (86Fa), UAS-FLP/TM6b*
		TO *y, w, hs-FLP1.22; UAS-mCD8::GFP, Mhc-RFP/CyO; PBac{w[+mC]=IT.GAL4}2090-G4/TM6b*
Fig. 4G	unpg memory system	Stock *y, w, hs-FLP1.22; TI{GFP[3xP3.cLa]=CRIMIC.TG4.1}unpg[CR00787-TG4.1], UAS-mCD8::GFP, Mhc-RFP/CyO; Mhc>>GAL4, UAS-FLP/TM6b*
Figs. 5 and 6	scRNA-seq 0 hours APF + 5 hours APF + 12 hours APF	*yw; UAS-mCD8::GFP//; Mef2-Gal4//*
		TO *Oregon-R-C*
Fig. 5F	tnc and twi labeling	*y, w, hs-FLP1.22; UAS-mCD8::GFP, Mhc-RFP/CyO; PBac{w[+mC]=IT.GAL4}2090-G4/TM6b*
		TO *y,w; twi-LacZ/CyO;+*
Fig. 5H	bi labeling	y, w, P{w[+m*]=GAL4}MD735;+;+
		TO *y, w, hs-FLP1.22; UAS-mCD8::GFP/CyO; TM6b/MKRS*
Fig. 5 (J and K)	tilm labeling	Stock *y, w, hs-FLP1.22; PBac{w[+mC]=IT.GAL4}RtGEF[4046-G4], UAS-mCD8::GFP, Mhc-RFP/CyO; MKRS/TM6b*
Fig. 5M	sv labeling	*y, w;TI{GFP[3xP3.cLa]=CRIMIC.TG4.2}sv[CR00370-TG4.2]*
		TO *y, w, hs-FLP1.22; UAS-mCD8::GFP/CyO; TM6b/MKRS*
Fig. 5N1	bi GAL80ts	y, w, P{w[+m*]=GAL4}MD735/FM7; UAS-mCD8::GFP, Mhc-RFP/CyO; MKRS/TM6b
		TO *P{w[+mC]=tubP-GAL80[ts]}Sxl[9], w /FM7c*
Fig. 5N2	tilm memory system	*y, w; Mhc>>GAL4(attP40), Mhc-RFP, UAS-mCD8::GFP/CyO; Mhc>>GAL4(86Fa), UAS-FLP/TM6b;+*
		TO *w[1118]; PBac{w[+mC]=IT.GAL4}RtGEF[4046-G4]/CyO*
Fig. 5N3	sv memory system	*y, w; Mhc>>GAL4(attP40), Mhc-RFP, UAS-mCD8::GFP/CyO; Mhc>>GAL4(86Fa), UAS-FLP/TM6b*
		TO *y, w; +; +; TI{GFP[3xP3.cLa]=CRIMIC.TG4.2}sv[CR00370-TG4.2]*
Fig. 6	Twi labeling	*y, w, hs-FLP1.22; UAS-mCD8::GFP, Mhc-RFP/CyO; PBac{w[+mC]=IT.GAL4}2090-G4/TM6b*
		TO *y, w; twi-LacZ/CyO;+*
Fig. 6 (F to H)	tilm labeling	Stock *y, w, hs-FLP1.22; PBac{w[+mC]=IT.GAL4}RtGEF[4046-G4], UAS-mCD8::GFP, Mhc-RFP/CyO; MKRS/TM6b*
Fig. 7D	Control RNAi	Stock *y, w; Mhc-GAL4, UAS-mCD8::GFP,Mhc-RFP/CyO; zfh1-T2A-GAL4, UAS-Dicer-2/TM6b*
Fig. 7E	RNAi bi	*y, w; Mhc-GAL4, UAS-mCD8::GFP,Mhc-RFP/CyO; zfh1-T2A-GAL4, UAS-Dicer-2/TM6b*
		TO *P{KK108384}VIE-260B*
Fig. 7F	RNAi Lim1	*y, w; Mhc-GAL4, UAS-mCD8::GFP,Mhc-RFP/CyO; zfh1-T2A-GAL4, UAS-Dicer-2/TM6b*
		TO *y, w; P{KK109046}VIE-260B/CyO; P{y[+t7.7] v[+t1.8]=TRiP.JF02503}attP2/TM6b*
Fig. 7H	RNAi Msh/Dr	*y, w; Mhc-GAL4, UAS-mCD8::GFP,Mhc-RFP/CyO; zfh1-T2A-GAL4, UAS-Dicer-2/TM6b*
		TO *P{KK107967}VIE-260B*
Fig. 8A	tnc labeling	Stock *y, w, hs-FLP1.22; UAS-mCD8::GFP, Mhc-RFP/CyO; PBac{w[+mC]=IT.GAL4}2090-G4/TM6b*
Fig. 8C	Control dpp, tnc readout	*y, w; dpp[hr56], twi-lacZ/SM6a, dfd-eYFP;+*
		TO *w; dpp[hr4], UAS-mCD8::GFP, Mhc-RFP/SM6-YFP; PBac{w[+mC]=IT.GAL4}2090-G4*
Fig. 8D	Mutant dpp, tnc readout	*y, w; dpp[hr56], twi-lacZ/SM6a, dfd-eYFP;+*
		TO *w; dpp[hr4], UAS-mCD8::GFP, Mhc-RFP/SM6-YFP; PBac{w[+mC]=IT.GAL4}2090-G4*
Fig. 8E	Control dpp, unpg readout	*y; dpp[hr56], twi-lacZ, bw/CyO, Tb;+*
		TO *w/+; dpp[hr4], TI{GFP[3xP3.cLa]=CRIMIC.TG4.1}unpg[CR00787-TG4.1], UAS-mCD8::GFP, Mhc-RFP/CyO, Tb;+*
Fig. 8F	Mutant dpp, unpg readout	*y; dpp[hr56], twi-lacZ, bw/CyO, Tb;+*
		TO *w/+; dpp[hr4], TI{GFP[3xP3.cLa]=CRIMIC.TG4.1}unpg[CR00787-TG4.1], UAS-mCD8::GFP, Mhc-RFP/CyO, Tb;+*
Fig. 8G	Control wg, tnc readout	*w; wg[l-12],twi-lacZ/SM6a, dfd-eYFP;PBac{w[+mC]=IT.GAL4}2090-G4/TM6b, Sb, dfd-eYFP*
		TO *w; wg[l-12],cn[1],bw[1]/SM6a, dfd-eYFP; UAS-mCD8::GFP/TM6b, Sb, dfd-eYFP*
Fig. 8H	Mutant wg, tnc readout	*w; wg[l-12],twi-lacZ/SM6a, dfd-eYFP;PBac{w[+mC]=IT.GAL4}2090-G4/TM6b, Sb, dfd-eYFP*
		TO *w; wg[l-12],cn[1],bw[1]/SM6a, dfd-eYFP; UAS-mCD8::GFP/TM6b, Sb, dfd-eYFP*
Fig. 8I	Control wg, unpg readout	*w; wg[l-12],cn[1],UAS-mCD8::GFP/CyO, Tb;+*
		TO *w; wg[l-12], TI{GFP[3xP3.cLa]=CRIMIC.TG4.1}unpg[CR00787-TG4.1]/CyO, bw, Tb;+*
Fig. 8J	Mutant wg, unpg readout	*w; wg[l-12],cn[1],UAS-mCD8::GFP/CyO, Tb;+*
		TO *w; wg[l-12], TI{GFP[3xP3.cLa]=CRIMIC.TG4.1}unpg[CR00787-TG4.1]/CyO, bw, Tb;+*
Fig. 9A	Control RNAi	Stock *y, w; Mhc-GAL4, UAS-mCD8::GFP,Mhc-RFP/CyO; zfh1-T2A-GAL4/TM6b*
Fig. 9B	RNAi Arm	*y, w; Mhc-GAL4, UAS-mCD8::GFP,Mhc-RFP/CyO; zfh1-T2A-GAL4/TM6b*
		TO *y, v; P{y[+t7.7] v[+t1.8]=TRiP.JF01252}attP2*
Fig. 9C	RNAi Dsh	*y, w; Mhc-GAL4, UAS-mCD8::GFP,Mhc-RFP/CyO; zfh1-T2A-GAL4/TM6b*
		TO *y, sc, v, sev; P{y[+t7.7], v[+t1.8]=TRiP.HMC05863}attP40*
Fig. 9D	RNAi Mad	*y, w; Mhc-GAL4, UAS-mCD8::GFP,Mhc-RFP/CyO; zfh1-T2A-GAL4/TM6b*
		TO *w; P{GD4121}v12635/CyO; UAS-Dicer-2/TM6b*
Fig. 10A	tilm labeling	Stock *y, w, hs-FLP1.22; PBac{w[+mC]=IT.GAL4}RtGEF[4046-G4], UAS-mCD8::GFP, Mhc-RFP/CyO; MKRS/TM6b*
Fig. 10C	Control wg, tilm readout	*w; wg[l-12],cn[1],bw[1]/CyO, bw, Tb;+*
		TO *w; wg[l-12], PBac{w[+mC]=IT.GAL4}RtGEF[4046-G4], UAS-mCD8::GFP, Mhc-RFP/CyO, Tb-RFP;Mhc>>GAL4(86Fa), UAS-FLP//*
Fig. 10D	Mutant wg, tilm readout	*w; wg[l-12],cn[1],bw[1]/CyO, bw, Tb;+*
		TO *w; wg[l-12], PBac{w[+mC]=IT.GAL4}RtGEF[4046-G4], UAS-mCD8::GFP, Mhc-RFP/CyO, Tb-RFP;Mhc>>GAL4(86Fa), UAS-FLP//*
Fig. 10I	Control wg, tilm readout adult	*w; wg[l-12], PBac{w[+mC]=IT.GAL4}RtGEF[4046-G4], UAS-mCD8::GFP, Mhc-RFP/CyO, Tb-RFP;Mhc>>GAL4(86Fa), UAS-FLP//*
		TO *w; wg[l-12],cn[1],bw[1]/CyO, bw, Tb; Mhc>>GAL4(86Fa), UAS-FLP/TM3*
Fig. 10J	Mutant wg, tilm readout adult	*w; wg[l-12], PBac{w[+mC]=IT.GAL4}RtGEF[4046-G4], UAS-mCD8::GFP, Mhc-RFP/CyO, Tb-RFP;Mhc>>GAL4(86Fa), UAS-FLP//*
		TO *w; wg[l-12],cn[1],bw[1]/CyO, bw, Tb; Mhc>>GAL4(86Fa), UAS-FLP/TM3*
Fig. 10K	Control dpp, tilm readout	*y, w; dpp[hr56], twi-lacZ, bw/CyO, Tb;+*
		TO *w; dpp[hr4], PBac{w[+mC]=IT.GAL4}RtGEF[4046-G4], UAS-mCD8::GFP, Mhc-RFP/CyO, bw;+*
Fig. 10 (L and M)	Mutant dpp, tilm readout	*y, w; dpp[hr56], twi-lacZ, bw/CyO, Tb;+*
		TO *w; dpp[hr4], PBac{w[+mC]=IT.GAL4}RtGEF[4046-G4], UAS-mCD8::GFP, Mhc-RFP/CyO, bw;+*

### Method details

#### RNAi experiments

Crosses were performed at 25°C, and the progeny were shifted to 29°C at 24 hours AEL. Adult male and female legs were dissected. Because both sexes displayed similar phenotypes, only T1 legs from males are shown in this study.

#### Leg imaging

Legs were dissected, fixed, and imaged as described by Guan *et al.* (*43*). We noticed that the internal tendon fluoresces at the same wavelength as the cuticle. To visualize the tendon, the channel corresponding to cuticle autofluorescence was isolated, and all external cuticle signals were manually removed using ImageJ software (version 2.16), leaving only the tendon autofluorescence (pink in Fig. 7).

#### Imaging of prothorax with attached forelegs

Heads, abdomens, and midleg and hind leg were removed in 1× phosphate-buffered saline (PBS). The thorax, with attached forelegs, was cut along the midline using scissors to obtain two hemi-thoraces. Distal tarsi were removed to improve fixation. Fixation was performed in 4% formaldehyde in 1× PBS for 30 min at room temperature. Imaging was performed as described by Guan *et al.* (*43*).

#### Quantification of muscle volume

The images of *Drosophila* legs were acquired using a Leica SP8 STED microscope with a 20× objective. After acquisition, the Lightning option in the Leica software was applied to improve the contrast and resolution of fluorescence images. The resulting *z*-stacks were preprocessed in Fiji (ImageJ) to reduce autofluorescence signal of cuticle in muscle channel, and the volumes of the tibia levator muscle (*tilm*) and femur muscles were quantified using the Surface module in Imaris. At least 10 legs per genotype were analyzed.

#### Immunostaining of embryos

Embryos were collected overnight (16 hours) on apple juice agar plates with yeast. They were dechorionated in bleach for 3 min, washed with water, and then fixed for 45 min in a solution of 500 μl of 4% formaldehyde and 500 μl of heptane.

Following fixation, embryos were transferred onto double-sided adhesive tape. The heptane was fully removed, and embryos are left to air dry under a fume hood for 5 min to allow strong adhesion.

Next, 0.1% PBST (PBS with 0.1% Triton X-100) was added, and embryos were manually devitellinized using a tungsten needle. A dorsal midline incision was made starting at the posterior end, and gentle pressure was applied to the anterior pole to expel the embryo.

Last, embryos were transferred into 1.5 ml of PBST and processed for immunostaining, following a protocol similar to that used for larval or pupal samples.

#### Immunostaining of leg disc during larval and pupal stages

Larvae and pupae were placed in ice-cold 1× PBS in a dissection dish to reduce movement. After a quick rinse with PBS, larvae and young pupae (0 hours APF) were reverted by cutting the distal region and turning them inside out to expose internal tissues. For pupae at 5 hours APF, cuticle, head and abdomen were first removed, and, then, a dorsal midline incision was made to allow penetration of the fixative. The fat body and gut were removed.

Immediately after dissection, reverted larvae or opened pupae were transferred to ice-cold 4% formaldehyde freshly prepared in PBS.

Once ~10 reverted larvae or opened pupae were in the cold fixative, the samples were moved to room temperature for fixation: 20 min for larvae and 25 min for pupae. Before antibody incubation, samples were washed five times (20 min each) with PBST-BSA (PBS with 0.3% Triton X-100 and 1% bovine serum albumin).

After washing, leg discs from larvae or pupae were dissected and incubated in PBST-BSA for 1 hour at room temperature. Samples were then incubated with primary antibodies diluted in PBST-BSA at 4°C overnight with gentle shaking.

Following incubation, samples were washed five times (20 min each) with PBST-BSA and then incubated with secondary antibodies (diluted in PBST-BSA) at 4°C overnight with gentle shaking. A final series of five washes (20 min each) was performed with PBST. Last, samples were mounted on glass slides using Vectashield antifade mounting medium (Vector Laboratories), and slides were imaged immediately.

#### Dilutions of primary and secondary antibodies

All secondary antibodies listed in the resource viability table were diluted at 1:500. Primary antibodies were diluted as follows: mouse anti–β-galactosidase (1/500), chicken anti–β-galactosidase (1/500), mouse anti-Dachshund (1/100), mouse anti–Fasciclin III (1/100), rabbit anti-Bi(omb) (1/1000), rabbit anti-Doc2 (1/1000), rabbit anti-Dpp (1/100), mouse anti-En (1/2), rabbit anti-Hth (1/5000), mouse anti-Lbe (1/500), rabbit anti-Lim1 (1/50), rabbit anti-Mef2 (1/750), rabbit anti-Msh (=Dr) (1/500), rabbit anti-Sox100B (1/1000, preabsorbed), rabbit anti-Tenectin (1/3000), rabbit anti-Twist (1/500), mouse anti-Wg (1/50), guinea pig anti-Dll (1/1000), and Phalloïdin Alexa 546 (1/500).

#### Random lineage tracing of MPs using the Flybow system

The Flybow system was used (UAS-Flybow 1.1) to randomly label MPs and trace their lineage through to the adult stage. The UAS-Flybow 1.1 construct enables stochastic expression of fluorescent proteins from a single transgene via excision events mediated by the heat shock–inducible *hs-FLP5* recombinase. To drive expression of the transgenes, *Mef2-GAL4* was used. In the absence of recombination, the GFP transgene is located adjacent to the upstream activating sequence (UAS) sequences in the correct orientation, resulting in uniform GFP expression in all adult muscles. For lineage analysis, mCherry^+^ clones were specifically tracked, which were readily identifiable using a fluorescence stereomicroscope. To ensure that only single recombination events were visualized, experimental conditions were optimized to achieve a low clone induction frequency, with ~1.5% of clones observed in the prothoracic segment. Heat shocks were performed at 37°C for 40 min at 24 hours AEL; 30 min at 48, 72, and 84 hours AEL; and 35 min at 96 hours AEL.

#### Dissection of leg disc for scRNA-seq

scRNA-seq was conducted on GFP^+^ MPs/myoblasts at different time points during development: 93 to 96 hours AEL (mid-L3 stage), 108 to 111 hours AEL, 0 hours APF (white pupae), 5 hours APF, and 12 hours APF. For each single-cell preparation, a pool of leg discs was collected from the different developmental time points. MPs/myoblasts were labeled with a membrane GFP (*UAS-mCD8::GFP*) under the control of *24B-GAL4* enhancer trap transgene for earlier time points (93 to 96 hours AEL and 108 to 111 hours AEL) that is expressed in MPs at early time points or *Mef2-GAL4* transgene for the later points time points (0 hours APF, 5 hours APF, and 12 hours APF) (*Mef2-GAL4* is a transgene containing the regulatory region of the myocyte enhancer factor 2 gene). Crosses between Oregon-R-C strain with the different stocks (*24B>GFP* or *Mef2>GFP*) were performed on day 1, and, the next day, an egg collection of 3 hours was made, and the flies were raised at 25°C. Subsequently, T1 leg discs were dissected in less than 1 hour to ensure tissue integrity, in M3 medium. Then, they were transferred, using a glass Pasteur pipette, into a 1.5-ml tube with 450 μl of M3 medium. Number of discs dissected per developmental stages: 48 discs at 93 to 96 hours AEL, 96 discs at 108 to 111 hours AEL, 82 discs at 0 hours APF, 80 discs at 5 hours APF, and 80 discs at 12 hours APF.

#### Cell dissociation before single-cell sequencing

The protocol was adapted from Harzer *et al.* (*44*). We added 25 μl of collagenase I and 25 μl of papain to a tube containing M3 medium and the leg discs (ensure the discs have sunk to the bottom of the tube). The tube was then placed in a Thermomixer and incubated for 1 hour at 30°C with shaking at 300 rpm. During the incubation, the contents were gently mixed twice by aspirating 200 μl of the dissociation solution and expelling it forcefully.

After incubation, the dissociation solution was carefully removed without disturbing the discs at the bottom of the tube, ensuring that all discs had sunk. Then, 500 μl of 1× PBS and 5% fetal bovine serum (FBS) was added, and the leg discs were mechanically dissociated using a 200-μl pipette tip by pipetting up and down 10 to 20 times until the solution appeared homogeneous. Last, the tubes were kept on ice until the FACS step.

#### FACS before single-cell sequencing

Fluorescence-activated cell sorting (FACS) was performed on a BD FACSAria II (AniRA-Cytometry platform, SFR Biosciences). Dead cells were excluded by adding 4′,6-diamidino-2-phenylindole (DAPI) to the sample, and high-quality single cells were sorted into cold 1× PBS supplemented with 5% FBS. Cytometry data were analyzed using BD FACS Diva software version 9.0.1.

Number of cells isolated by FACS: 5979 cells at 93 to 96 hours AEL, 15,543 cells at 108 to 111 hours AEL, 25,872 cells at 0 hours APF, 32,934 cells at 5 hours APF, and 19,000 cells at 12 hours APF.

After the isolation of GFP^+^ cells by FACS, the sample is centrifuged for 5 min at 500*g* at 4°C (2017 rpm = 500 relative centrifugal force), and the volume was calculated depending on the number of cells needed for the sequencing experiment. The appropriate supernatant volume was removed, and the pellet was resuspended in the adequate volume. A control of cell concentration using a Malassez chamber was done before the library preparation.

#### Library preparation (10x Genomics protocol)

Preparation of the scRNA library was performed using the 10x Genomics single-cell capturing system. Cell suspensions were loaded onto the 10x Genomics Chromium Controller following the manufacturer’s instructions. Then, single-cell cDNA libraries were prepared using the Chromium Single Cell 3′ Reagent Kit version v3.1. For all the time points, a simple index sequence is used, except for time point 2 (108 to 111 hours AEL) where a dual index is used. Before the sequencing step, the amount, size, and quality of the cDNA were assessed using the TapeStation 2200 (Agilent DNA ScreenTape High Sensitivity System) and the Qubit system.

#### Single-cell sequencing

The resulting libraries were sequenced by the Institut de Génomique Fonctionnelle de Lyon (IGFL)’s sequencing platform (PSI, Lyon, France) using an Illumina NextSeq 500 sequencer [28 base pairs (bp) for R1 and 132 bp for R2].

*Data preprocessing*Raw sequencing data were generated in base call format (BCL), which contains data for all libraries within the sequencing run. These BCL files were converted into FASTQ format using the makefastq command from the Cell Ranger software (version 5.0.0; 10x Genomics) (*45*). The Cell Ranger pipeline enables demultiplexing of BCL files into FASTQ files corresponding to individual libraries.

Once FASTQ files were generated for each sample, the Cell Ranger pipeline was used for alignment of reads to the reference genome and unique molecular identifier (UMI) counting. Reads were mapped to a customized *D. melanogaster* reference genome (dm6, NCBI RefSeq assembly GCF_000001215.4), modified to include the *UAS-mCD8::GFP* transgene sequence to ensure accurate alignment of reads derived from this construct.

*Single-cell RNA-seq data analysis with Seurat V4*Single-cell RNA-seq data at 93 to 96 hours AEL were analyzed using the Seurat R package (version 4) (*46*), which facilitates quality control (QC), normalization, integration, and exploration of single-cell transcriptomic data. Seurat is specifically designed to identify and interpret sources of cellular heterogeneity and supports integration of diverse single-cell datasets. Upon loading the data, a Seurat object was created, and initial QC metrics were assessed. Cells were filtered based on the following criteria:

1) Genes expressed in fewer than three cells were excluded (parameter min.cells) to remove uninformative genes.

2) Cells with fewer than 200 detected genes were removed (parameter min.features) as these typically represent low-quality cells or empty droplets.

3) Cells with total UMI counts of >150,000 (or >100,000 for 0 hours APF and 12 hours APF) or <0 were excluded to remove potential doublets or technical artifacts.

4) Cells with >5% mitochondrial gene content were excluded as high mitochondrial content often indicates stressed or dying cells (mitochondrial genes were identified as those beginning with “MT:”).

5) Only GFP^+^ and *twi*^+^ cells were retained.

Following cell filtering, data normalization was performed using Seurat’s SCTransform workflow (*47*). Highly variable genes, those with substantial expression variability across cells, were identified for downstream analysis. A linear transformation (principal components analysis) was applied to reduce dimensionality before further processing.

Subsequently, clustering was performed, followed by nonlinear dimensionality reduction using UMAP to visualize and explore the data structure. Stochastic neighbor embedding methods aim to project cells into a low-dimensional space while trying to preserve local neighborhood structures, such that cells within the same cluster are positioned close to one another.

Last, Seurat was used to identify marker genes defining each cluster through differential expression analysis. By default, Seurat identifies both positive and negative markers for a given cluster compared to all other cells. This analysis can also be extended to compare groups of clusters or perform global comparisons across the dataset.

*Single-cell RNA-seq data analysis with Scanpy*In parallel to the Seurat workflow, an alternative analysis strategy was implemented using the Scanpy toolkit (*48*) to integrate all samples and perform downstream single-cell RNA-seq analysis. Datasets from multiple time points (93 to 96 hours AEL, 108 to 111 hours AEL, 0 hours APF, 5 hours APF, and 12 hours APF) were combined into a single integrated dataset for joint analysis.

The initial preprocessing steps involved filtering out low-quality cells and uninformative genes Cells with mitochondrial gene percentages exceeding 20% were excluded to remove low-quality or dying cells. Additional filtering criteria removed cells with fewer than 600 or more than 5000 detected genes, and cells with total counts above 150,000. Genes expressed in fewer than three cells were also removed. For specific developmental stages, targeted filtering was applied: At 93 to 96 hours AEL and 108 to 111 hours AEL, only cells expressing at least one of GFP or Twi were kept; at 0 hours APF, 5 hours APF, and 12 hours APF, only cells expressing at least one of the following genes: GFP, *Mef2*, or *twi*, were retained.

Filtered data were normalized by scaling each cell to a total of 10,000 counts. The normalized counts were then log-transformed [log(*x* + 1)] to stabilize variance for downstream analysis. Last, gene expression values were scaled to unit variance and zero mean.

Batch effects across samples were corrected using the Batch Balanced K Nearest Neighbours (BBKNN) algorithm (*49*), which preserves the local structure of the data while adjusting for technical variability between batches. A first analysis and clustering were performed on each time point individually, and differential gene expression analysis was conducted similarly to the Seurat-based workflow to identify marker genes associated with each cluster. Further analysis was conducted on the combined dataset with all the time points.

*Single-cell RNA-seq pseudotime analysis with PAGA*To investigate the temporal evolution of cellular states and connect different developmental time points, pseudotime analysis was performed using the Partition-based Graph Abstraction (PAGA) algorithm implemented in Scanpy (*22*). PAGA constructed a graph-based representation of cellular trajectories, providing insight into the connectivity and progression between clusters within a heterogeneous cell population. This approach facilitates the reconstruction of lineage relationships and the temporal ordering of cells across developmental stages.

PAGA analysis was applied to a subset of the data including clusters 4, 6, 11, 13, 14_1, 14_2, and 15 (Fig. 5), which represent central MPs/myoblasts contributing to the development of telopodite muscles. Cluster 11, corresponding to central MPs at the earliest time point, was selected as the root for pseudotime inference (Figs. 5 and 7 and figs. S5 and S6).

PAGA connectivities tree and ForceAtlas2 (FA2) map pseudotime color coded were generated across all central MP/myoblast clusters. Lineages were defined using the following criteria: the strongest connection between clusters in PAGA connectivities tree graph; the shortest path in the PAGA connectivities tree graph between clusters; and coherent directionality observed in the FA2 map colored by normalized pseudotime.

To refine lineage directionality, pseudotime was also visualized on individual lineages, allowing us to distinguish trajectories that evolve at different speeds. Of note, cluster 14 was further subclustered, into 14_1 and 14_2, to improve resolution of the lineage leading to tibial and femoral muscles.

Last, clusters were color coded on the FA2 map using pseudotime-matched color gradients. This visualization revealed consistent temporal dynamics across lineages, supporting the conclusion that normalized pseudotime accurately reflects the developmental progression of central MPs.

#### Temporal induction of wg and dpp loss of function

Thermosensitive alleles were used for both *dpp* and *wg* experiments. The allele *wg[IL114]*, referred to here as *wg[l-12]*, was described by Van den Heuvel *et al.* (*50*). The thermosensitivity of the heteroallelic combination *dpp[hr4]/dpp[hr56]* was reported by Hsiung *et al.* (*51*), with the molecular nature of *hr56* and *hr4* detailed by Wharton *et al.* (*52*). To facilitate genotype preparation, polymerase chain reaction assays were developed to distinguish both *wg[l-12]* and *dpp[hr4]* alleles, which enabled to sort recombinant chromosomes efficiently. The *wg* mutant chromosome obtained from the Bloomington stock was homozygous lethal; through recombination, the lethal mutation was separated from the *wg* allele. Mutant crosses were maintained at the respective restrictive temperatures, 25°C for *wg* and 29°C for *dpp*, with control groups kept at 16°C. Egg collections were performed in 3 to 4 hours intervals, and developmental timing was adjusted accordingly to temperature to ensure that embryos and larvae reached the appropriate developmental stages before temperature shifts were applied (see Figs. 8 and 10 and fig. S10). Mutant animals were sorted on the presence of absence of Tb phenotype when using Tb marked balancer chromosome (*53*) or with yellow fluorescent protein marked balancer chromosome (*54*).

*dpp[hr4]/dpp[hr56]* or *wg[l-12]* samples were shifted to the nonpermissive temperature either before or after the specification of central versus peripheral MPs and dissected at the relevant stage wandering larvae or adults.

Shift before specification: Dpp mutant condition: 3 days AEL at 16°C; Wg mutant condition: 6 to 7 days AEL at 16°C. Shift after specification: Wg mutant condition: 10 days AEL at 16°C; Dpp mutant condition: 8 days and 14 hours AEL (early time point) or 9 days AEL (late time point) at 16°C. To obtain the equivalent developmental time for flies raised at 25°C, the time spent at 16°C must be divided by three.

#### Image acquisition

For immunostained leg discs, multiple 0.5-μm-thick sections in the *z* axis were imaged with a Leica SP8 confocal microscope using a 40× glycerol objective. Multiple 1.0-μm-thick sections in the *z* axis for adult legs were images with a Leica SP8 or a Zeiss LSM780 confocal microscope, using 20× or 25× glycerol objectives. A fluorescent stereomicroscope Leica M205 FA was used to select cherry positive clones (Fig. 3)

#### Software

Confocal pictures were processed and analyzed with ImageJ software (version 2.16), and Imaris software (version 10.2). Three-dimensional (3D) reconstructions were performed using Amira 3D software (version 6.2). Graphs and statistical visualizations were generated using Prism 10 (version 10.4.0). scRNA-seq data were processed with Cell Ranger (version 5.0.0) and analyzed using Seurat (version 4.1.1, R-based version 4.2.3) and Scanpy (version 1.9.1, Python-based version 3.10.11).

#### Schematics

All schematics were done with Microsoft PowerPoint.

#### Cloning

*Pattb-MHC-DSCP-5ʹFRT-stop-3ʹFRT-GAL4-hsp70*The *MHC* enhancer (*55*) was excised using Acc 65I (5′) and Xma I (3′) from a Bluescript vector (a gift from the Frank Schnorrer Lab) and cloned into a *Pattb-GAL4-hsp70* vector (a gift from the Richard S. Mann Lab) in front of the *GAL4* sequence, which had been opened with Nhe I (5′) and Sbf I (3′), resulting in the *Pattb-MHC-GAL4-hsp70* vector. In a second step, a *5′FRT-stop-3′FRT* cassette was excised from another vector (a gift from the Gary Struhl Lab) using Kpn I and inserted between the *MHC* enhancer and the GAL4 cassette in the *Pattb-MHC-GAL4-hsp70* vector, which had been opened with Kpn I. The resulting *Pattb-MHC-DSCP-5′FRT-stop-3′FRT-GAL4-hsp70* construct was inserted into chromosomes II and III by injection into embryos carrying the attP40 or attP86F landing sites.

#### Statistic

In (Fig. 7), muscle volume was quantified for each biological replicate and compared between experimental conditions using a two-tailed unpaired Student’s *t* test, as data were continuous, approximately normally distributed, and variances between groups were comparable. In (Fig. 10), we also use a two-tailed unpaired Student’s *t* test. In (Fig. 8), cell numbers between conditions were compared using a nonparametric Mann-Whitney *U* test (Wilcoxon rank sum test) with a one-sided alternative hypothesis (condition 1 < condition 2) as data consisted of nonnormally distributed cell counts with unequal variances. In (Fig. 8), frequency values between conditions were compared using a nonparametric Mann-Whitney *U* test as data consisted of bounded proportions and did not meet normality assumptions.

## References

[1] L. Solomon, D. Wa, Eds., *Apley’s System of Orthopaedics and Fractures* (CRC Press, Taylor & Francis); www.taylorfrancis.com/books/edit/10.1201/b13422/apley-system-orthopaedics-fractures-louis-solomon-david-warwick-selvadurai-nayagam.

[2] C. Soler, M. Daczewska, J. P. D. Ponte, B. Dastugue, K. Jagla, Coordinated development of muscles and tendons of the *Drosophila* leg. Development 131, 6041–6051 (2004). 15537687 10.1242/dev.01527

[3] P. A. Lawrence, Cell lineage of the thoracic muscles of Drosophila. Cell 29, 493–503 (1982). 7116447 10.1016/0092-8674(82)90166-0

[4] P. Schlegel, Y. Yin, A. S. Bates, S. Dorkenwald, K. Eichler, P. Brooks, D. S. Han, M. Gkantia, M. dos Santos, E. J. Munnelly, G. Badalamente, L. Serratosa Capdevila, V. A. Sane, A. M. C. Fragniere, L. Kiassat, M. W. Pleijzier, T. Stürner, I. F. M. Tamimi, C. R. Dunne, I. Salgarella, A. Javier, S. Fang, E. Perlman, T. Kazimiers, S. R. Jagannathan, A. Matsliah, A. R. Sterling, S. Yu, C. E. McKellar, M. Costa, H. S. Seung, M. Murthy, V. Hartenstein, D. D. Bock, G. S. X. E. Jefferis, Whole-brain annotation and multi-connectome cell typing of *Drosophila*. Nature 634, 139–152 (2024). 39358521 10.1038/s41586-024-07686-5 PMC11446831

[5] J. S. Phelps, D. G. C. Hildebrand, B. J. Graham, A. T. Kuan, L. A. Thomas, T. M. Nguyen, J. Buhmann, A. W. Azevedo, A. Sustar, S. Agrawal, M. Liu, B. L. Shanny, J. Funke, J. C. Tuthill, W.-C. A. Lee, Reconstruction of motor control circuits in adult *Drosophila* using automated transmission electron microscopy. Cell 184, 759–774.e18 (2021). 33400916 10.1016/j.cell.2020.12.013 PMC8312698

[6] M. Murphy, G. Kardon, Origin of vertebrate limb muscle: The role of progenitor and myoblast populations. Curr. Top. Dev. Biol. 96, 1–32 (2011). 21621065 10.1016/B978-0-12-385940-2.00001-2 PMC5573708

[7] B. Christ, B. Brand-Saberi, Limb muscle development. Int. J. Dev. Biol. 46, 905–914 (2002). 12455628

[8] F. Helmbacher, S. Stricker, Tissue cross talks governing limb muscle development and regeneration. Semin. Cell Dev. Biol. 104, 14–30 (2020). 32517852 10.1016/j.semcdb.2020.05.005

[9] P. Hasson, A. DeLaurier, M. Bennett, E. Grigorieva, L. A. Naiche, V. E. Papaioannou, T. J. Mohun, M. P. Logan, Tbx4 and Tbx5 acting in connective tissue are required for limb muscle and tendon patterning. Dev. Cell 18, 148–156 (2010). 20152185 10.1016/j.devcel.2009.11.013 PMC3034643

[10] M. P. Colasanto, S. Eyal, P. Mohassel, M. Bamshad, C. G. Bonnemann, E. Zelzer, A. M. Moon, G. Kardon, Development of a subset of forelimb muscles and their attachment sites requires the ulnar-mammary syndrome gene Tbx3. Dis. Model. Mech. 9, 1257–1269 (2016). 27491074 10.1242/dmm.025874 PMC5117227

[11] G. Kardon, B. D. Harfe, C. J. Tabin, A Tcf4-positive mesodermal population provides a prepattern for vertebrate limb muscle patterning. Dev. Cell 5, 937–944 (2003). 14667415 10.1016/s1534-5807(03)00360-5

[12] M. Buckingham, F. Relaix, PAX3 and PAX7 as upstream regulators of myogenesis. Semin. Cell Dev. Biol. 44, 115–125 (2015). 26424495 10.1016/j.semcdb.2015.09.017

[13] F. Relaix, D. Rocancourt, A. Mansouri, M. Buckingham, A Pax3/Pax7-dependent population of skeletal muscle progenitor cells. Nature 435, 948–953 (2005). 15843801 10.1038/nature03594

[14] K. Jagla, P. Dollé, M. G. Mattei, T. Jagla, B. Schuhbaur, G. Dretzen, F. Bellard, M. Bellard, Mouse Lbx1 and human LBX1 define a novel mammalian homeobox gene family related to the *Drosophila* lady bird genes. Mech. Dev. 53, 345–356 (1995). 8645601 10.1016/0925-4773(95)00450-5

[15] B. S. Mankoo, N. S. Collins, P. Ashby, E. Grigorieva, L. H. Pevny, A. Candia, C. V. Wright, P. W. Rigby, V. Pachnis, Mox2 is a component of the genetic hierarchy controlling limb muscle development. Nature 400, 69–73 (1999). 10403250 10.1038/21892

[16] M. E. Pownall, M. K. Gustafsson, C. P. Emerson, Myogenic regulatory factors and the specification of muscle progenitors in vertebrate embryos. Annu. Rev. Cell Dev. Biol. 18, 747–783 (2002). 12142270 10.1146/annurev.cellbio.18.012502.105758

[17] B. L. Black, E. N. Olson, Transcriptional control of muscle development by myocyte enhancer factor-2 (MEF2) proteins. Annu. Rev. Cell Dev. Biol. 14, 167–196 (1998). 9891782 10.1146/annurev.cellbio.14.1.167

[18] M. Bate, E. Rushton, D. A. Currie, Cells with persistent twist expression are the embryonic precursors of adult muscles in *Drosophila*. Development 113, 79–89 (1991). 1765010 10.1242/dev.113.1.79

[19] N. J. Everetts, M. I. Worley, R. Yasutomi, N. Yosef, I. K. Hariharan, Single-cell transcriptomics of the *Drosophila* wing disc reveals instructive epithelium-to-myoblast interactions. eLife 10, e61276 (2021). 33749594 10.7554/eLife.61276 PMC8021398

[20] A. Patel, Y. Wu, X. Han, Y. Su, T. Maugel, H. Shroff, S. Roy, Cytonemes coordinate asymmetric signaling and organization in the *Drosophila* muscle progenitor niche. Nat. Commun. 13, 1185 (2022). 35246530 10.1038/s41467-022-28587-z PMC8897416

[21] M. P. Zappia, L. de Castro, M. M. Ariss, H. Jefferson, A. B. Islam, M. V. Frolov, A cell atlas of adult muscle precursors uncovers early events in fibre-type divergence in *Drosophila*. EMBO Rep. 21, e49555 (2020). 32815271 10.15252/embr.201949555 PMC7534622

[22] F. A. Wolf, F. K. Hamey, M. Plass, J. Solana, J. S. Dahlin, B. Göttgens, N. Rajewsky, L. Simon, F. J. Theis, PAGA: Graph abstraction reconciles clustering with trajectory inference through a topology preserving map of single cells. Genome Biol. 20, 59 (2019). 30890159 10.1186/s13059-019-1663-x PMC6425583

[23] B. R. Hopkins, O. Barmina, A. Kopp, A single-cell atlas of the sexually dimorphic *Drosophila* foreleg and its sensory organs during development. PLOS Biol. 21, e3002148 (2023). 37379332 10.1371/journal.pbio.3002148 PMC10335707

[24] M. Ruiz-Losada, D. Blom-Dahl, S. Córdoba, C. Estella, Specification and patterning of *Drosophila* appendages. J. Dev. Biol. 6, 17 (2018). 30011921 10.3390/jdb6030017 PMC6162442

[25] C. Estella, R. S. Mann, Logic of Wg and Dpp induction of distal and medial fates in the *Drosophila* leg. Dev. Camb. Engl. 135, 627–636 (2008). 10.1242/dev.014670 PMC2820282 18184724

[26] G. Struhl, K. Basler, Organizing activity of wingless protein in Drosophila. Cell 72, 527–540 (1993). 8440019 10.1016/0092-8674(93)90072-x

[27] T. Isshiki, B. Pearson, S. Holbrook, C. Q. Doe, *Drosophila* neuroblasts sequentially express transcription factors which specify the temporal identity of their neuronal progeny. Cell 106, 511–521 (2001). 11525736 10.1016/s0092-8674(01)00465-2

[28] X. Li, Z. Chen, C. Desplan, Temporal patterning of neural progenitors in *Drosophila*. Curr. Top. Dev. Biol. 105, 69–96 (2013). 23962839 10.1016/B978-0-12-396968-2.00003-8 PMC3927947

[29] Z. Liu, C.-P. Yang, K. Sugino, C.-C. Fu, L.-Y. Liu, X. Yao, L. P. Lee, T. Lee, Opposing intrinsic temporal gradients guide neural stem cell production of varied neuronal fates. Science 350, 317–320 (2015). 26472907 10.1126/science.aad1886

[30] J. Delile, T. Rayon, M. Melchionda, A. Edwards, J. Briscoe, A. Sagner, A. Klein, B. Treutlein, Single cell transcriptomics reveals spatial and temporal dynamics of gene expression in the developing mouse spinal cord. Development 146, dev173807 (2019). 30846445 10.1242/dev.173807 PMC6602353

[31] S. M. Watt, M. G. Roubelakis, Deciphering the complexities of adult human steady state and stress-induced hematopoiesis: Progress and challenges. Int. J. Mol. Sci. 26, 671 (2025). 39859383 10.3390/ijms26020671 PMC11766050

[32] W. Guan, S. Bellemin, M. Bouchet, L. Venkatasubramanian, C. Guillermin, A. Laurençon, C. Kabir, A. Darnas, C. Godin, S. Urdy, R. S. Mann, J. Enriquez, Post-transcriptional regulation of transcription factor codes in immature neurons drives neuronal diversity. Cell Rep. 39, 110992 (2022). 35767953 10.1016/j.celrep.2022.110992 PMC9479746

[33] J. Enriquez, L. Venkatasubramanian, M. Baek, M. Peterson, U. Aghayeva, R. S. Mann, Specification of individual adult motor neuron morphologies by combinatorial transcription factor codes. Neuron 86, 955–970 (2015). 25959734 10.1016/j.neuron.2015.04.011 PMC4441546

[34] S. González-Crespo, G. Morata, Genetic evidence for the subdivision of the arthropod limb into coxopodite and telopodite. Development 122, 3921–3928 (1996). 9012512 10.1242/dev.122.12.3921

[35] G. Kardon, J. K. Campbell, C. J. Tabin, Local extrinsic signals determine muscle and endothelial cell fate and patterning in the vertebrate limb. Dev. Cell 3, 533–545 (2002). 12408805 10.1016/s1534-5807(02)00291-5

[36] M. Dos Santos, S. Backer, F. Auradé, M. M.-K. Wong, M. Wurmser, R. Pierre, F. Langa, M. Do Cruzeiro, A. Schmitt, J.-P. Concordet, A. Sotiropoulos, F. Jeffrey Dilworth, D. Noordermeer, F. Relaix, I. Sakakibara, P. Maire, A fast *Myosin* super enhancer dictates muscle fiber phenotype through competitive interactions with *Myosin* genes. Nat. Commun. 13, 1–17 (2022). 35210422 10.1038/s41467-022-28666-1 PMC8873246

[37] M. Yamamoto, A. Kuroiwa, Hoxa-11 and Hoxa-13 are involved in repression of MyoD during limb muscle development. Dev. Growth Differ. 45, 485–498 (2003). 14706073 10.1111/j.1440-169x.2003.00715.x

[38] K. Hashimoto, Y. Yokouchi, M. Yamamoto, A. Kuroiwa, Distinct signaling molecules control Hoxa-11 and Hoxa-13 expression in the muscle precursor and mesenchyme of the chick limb bud. Development 126, 2771–2783 (1999). 10331987 10.1242/dev.126.12.2771

[39] M. Yamamoto, Y. Gotoh, K. Tamura, M. Tanaka, A. Kawakami, H. Ide, A. Kuroiwa, Coordinated expression of Hoxa-11 and Hoxa-13 during limb muscle patterning. Development 125, 1325–1335 (1998). 9477331 10.1242/dev.125.7.1325

[40] H. Asfour, E. Hirsinger, R. Rouco, F. Zarrouki, S. Hayashi, S. Swist, T. Braun, K. Patel, F. Relaix, G. Andrey, S. Stricker, D. Duprez, A. Stantzou, H. Amthor, Inhibitory SMAD6 interferes with BMP-dependent generation of muscle progenitor cells and perturbs proximodistal pattern of murine limb muscles. Dev. Camb. Engl. 150, dev201504 (2023). 10.1242/dev.201504 37272529

[41] B. Alaniz, T. Alghamdi, H. Alhaji, H. Alghalaf, H. Aldossary, K. Aldajani, R. Alotaibi, S. S. Mohiuddin, A comprehensive review study on muscular dystrophy and its associated impact on health and individuals. Orthop. Muscular Syst. Curr. Res. 8, 1–6 (2018).

[42] A. E. Emery, The muscular dystrophies. Lancet 359, 687–695 (2002). 11879882 10.1016/S0140-6736(02)07815-7

[43] W. A. Guan, L. A. Venkatasubramanian, M. A. Baek, R. A. Mann, J. A. Enriquez, Visualize *Drosophila* leg motor neuron axons through the adult cuticle. J. Vis. Exp., 10.3791/58365 (2018). 10.3791/58365 PMC6544026 30451217

[44] H. Harzer, C. Berger, R. Conder, G. Schmauss, J. A. Knoblich, FACS purification of *Drosophila* larval neuroblasts for next-generation sequencing. Nat. Protoc. 8, 1088–1099 (2013). 23660757 10.1038/nprot.2013.062 PMC3930877

[45] G. X. Y. Zheng, J. M. Terry, P. Belgrader, P. Ryvkin, Z. W. Bent, R. Wilson, S. B. Ziraldo, T. D. Wheeler, G. P. McDermott, J. Zhu, M. T. Gregory, J. Shuga, L. Montesclaros, J. G. Underwood, D. A. Masquelier, S. Y. Nishimura, M. Schnall-Levin, P. W. Wyatt, C. M. Hindson, R. Bharadwaj, A. Wong, K. D. Ness, L. W. Beppu, H. J. Deeg, C. McFarland, K. R. Loeb, W. J. Valente, N. G. Ericson, E. A. Stevens, J. P. Radich, T. S. Mikkelsen, B. J. Hindson, J. H. Bielas, Massively parallel digital transcriptional profiling of single cells. Nat. Commun. 8, 14049 (2017). 28091601 10.1038/ncomms14049 PMC5241818

[46] Y. Hao, T. Stuart, M. H. Kowalski, S. Choudhary, P. Hoffman, A. Hartman, A. Srivastava, G. Molla, S. Madad, C. Fernandez-Granda, R. Satija, Dictionary learning for integrative, multimodal and scalable single-cell analysis. Nat. Biotechnol. 42, 293–304 (2024). 37231261 10.1038/s41587-023-01767-y PMC10928517

[47] C. Hafemeister, R. Satija, Normalization and variance stabilization of single-cell RNA-seq data using regularized negative binomial regression. Genome Biol. 20, 296 (2019). 31870423 10.1186/s13059-019-1874-1 PMC6927181

[48] F. A. Wolf, P. Angerer, F. J. Theis, SCANPY: Large-scale single-cell gene expression data analysis. Genome Biol. 19, 15 (2018). 29409532 10.1186/s13059-017-1382-0 PMC5802054

[49] K. Polański, M. D. Young, Z. Miao, K. B. Meyer, S. A. Teichmann, J.-E. Park, BBKNN: Fast batch alignment of single cell transcriptomes. Bioinformatics 36, 964–965 (2020). 31400197 10.1093/bioinformatics/btz625 PMC9883685

[50] M. van den Heuvel, C. Harryman-Samos, J. Klingensmith, N. Perrimon, R. Nusse, Mutations in the segment polarity genes wingless and porcupine impair secretion of the wingless protein. EMBO J. 12, 5293–5302 (1993). 8262072 10.1002/j.1460-2075.1993.tb06225.x PMC413795

[51] F. Hsiung, F.-A. Ramirez-Weber, D. D. Iwaki, T. B. Kornberg, Dependence of *Drosophila* wing imaginal disc cytonemes on Decapentaplegic. Nature 437, 560–563 (2005). 16177792 10.1038/nature03951

[52] K. Wharton, R. P. Ray, S. D. Findley, H. E. Duncan, W. M. Gelbart, Molecular lesions associated with alleles of decapentaplegic identify residues necessary for TGF-beta/BMP cell signaling in *Drosophila melanogaster*. Genetics 142, 493–505 (1996). 8852848 10.1093/genetics/142.2.493 PMC1206983

[53] C. Pina, F. Pignoni, Tubby-RFP balancers for developmental analysis: FM7c 2xTb-RFP, CyO 2xTb-RFP, and TM3 2xTb-RFP. Genesis 50, 119–123 (2012). 21913310 10.1002/dvg.20801 PMC3931234

[54] T. Le, Z. Liang, H. Patel, M. H. Yu, G. Sivasubramaniam, M. Slovitt, G. Tanentzapf, N. Mohanty, S. M. Paul, V. M. Wu, G. J. Beitel, New family of Drosophila balancer chromosomes with a *w*^−^ *dfd*-GMR yellow fluorescent protein marker. Genetics 174, 2255–2257 (2006). 17057238 10.1534/genetics.106.063461 PMC1698648

[55] E. H. Chen, E. N. Olson, Antisocial, an intracellular adaptor protein, is required for myoblast fusion in Drosophila. Dev. Cell 1, 705–715 (2001). 11709190 10.1016/s1534-5807(01)00084-3

[56] A. Miller, “The internal anatomy and histology of the imago of *Drosophila melanogaster*,” in *Biology of Drosophila* (Hafner Pub. Co., 1950), pp. 420–534; https://libcatalog.usc.edu/discovery/fulldisplay/alma991026817859703731/01USC_INST:01USC.

[57] C. A. Schneider, W. S. Rasband, K. W. Eliceiri, NIH Image to ImageJ: 25 Years of image analysis. Nat. Methods 9, 671–675 (2012). 22930834 10.1038/nmeth.2089 PMC5554542

[58] Y. Hao, S. Hao, E. Andersen-Nissen, W. M. Mauck, S. Zheng, A. Butler, M. J. Lee, A. J. Wilk, C. Darby, M. Zager, P. Hoffman, M. Stoeckius, E. Papalexi, E. P. Mimitou, J. Jain, A. Srivastava, T. Stuart, L. M. Fleming, B. Yeung, A. J. Rogers, J. M. McElrath, C. A. Blish, R. Gottardo, P. Smibert, R. Satija, Integrated analysis of multimodal single-cell data. Cell 184, 3573–3587.e29 (2021). 34062119 10.1016/j.cell.2021.04.048 PMC8238499

