# Supplementary material for: Spatiotemporal control of myoblast identity drives muscle diversity in the Drosophila leg
Source: Sci Adv. 2026 Jul 1;12(27):eaed0910. doi: 10.1126/sciadv.aed0910 (PMC13322264; doi:10.1126/sciadv.aed0910)
Supplement: Supplementary file 1 — Supplementary Text Figs. S1 to S10 Table S1 Legends for movies S1 and S2 [file sciadv.aed0910_sm.pdf]

Science Advances
AAAS

Supplementary Materials for

Spatiotemporal control of myoblast identity drives muscle diversity in the *Drosophila* leg

Camille Guillermin *et al.*

Corresponding author: Jonathan Enriquez, jonathan.enriquez@ens-lyon.fr

Sci. Adv. **12**, eaed0910 (2026)
DOI: 10.1126/sciadv.aed0910

The PDF file includes:

Supplementary Text
Figs. S1 to S10
Table S1
Legends for movies S1 and S2

Other Supplementary Material for this manuscript includes the following:

Movies S1 and S2

Supplementary Text

Genetic crosses for each supplementary figures:

**Figures**	**Summary**	**Genetic crosses**
Fig. S1	Mhc labeling	*y, w; Mhc-GAL4(attP40)/CyO; TM6b/MKRS* TO *y, w, hs-Flp1.22; UAS-mCD8::GFP/CyO; TM6b/MKRS*
Fig. S2B	Mhc labeling	*y, w; Mhc-GAL4(attP40)/CyO; TM6b/MKRS* TO *y, w, hs-Flp1.22; UAS-mCD8::GFP/CyO; TM6b/MKRS*
Fig. S2C	Mhc labeling	Stock *y, w; Mhc-GAL4, UAS-mCD8::GFP,Mhc-RFP/CyO; zfh1-T2A-GAL4/TM6b*
Fig. S3	Mef2 labeling	Stock *y, w; UAS-mCD8::GFP, Mhc-RFP/CyO; Mef2-GAL4/ TM6b*
Fig. S4A	unpg labeling	Stock *w ; TI{GFP[3xP3.cLa]=CRIMIC.TG4.1}unpg[CR00787-TG4.1], UAS-mCD8::GFP, Mhc-RFP/CyO ; MKRS/TM6b*
Fig. S4B	tnc labeling	Stock *y, w, hs-Flp1.22; UAS-mCD8::GFP, Mhc-RFP/CyO; PBac{w[+mC]=IT.GAL4}2090-G4/TM6b*
Fig. S4C	bi labeling	_y, w, P{w[+m*]=GAL4}MD735 ; + ; +_ TO *y, w, hs-Flp1.22; UAS-mCD8::GFP/CyO; TM6b/MKRS*
Fig. S4D	ko labeling	*y, w ; Mi{Trojan-GAL4.0}ko[MI10038-TG4.0]/TM3, Sb, Ser* TO *y, w, hs-Flp1.22/w; UAS-mCD8::GFP ; MKRS / SM5-TM6b*
Fig. S4E	Lim1 AD labeling	*w, Lim1-T2A-VP16/FM7;+;+* TO *y, w ; P{w[+mC]=Tub-GAL4DBD.D}2; P{w[+mC]=UAS-2xEGFP}AH3*
Fig. S4F	bi Flp and Mhc labeling	*y, w; Mhc>>Gal4(attP40), Mhc-RFP, UAS-mCD8::GFP/CyO; Mhc>>GAL4(86Fa), UAS-FLP/TM6b* TO _y, w, P{w[+m*]=GAL4}MD735;+;+_
Fig. S4G	Ko Flp and Mhc labeling	*y, w; Mhc>>Gal4(attP40), Mhc-RFP, UAS-mCD8::GFP/CyO; Mhc>>GAL4(86Fa), UAS-FLP/TM6b* TO *Mi{Trojan-GAL4.0}ko[MI10038-TG4.0]*
Fig. S5; Fig. S6; Fig. S9	ScRNAseq 0h APF + 5h APF + 12h APF	*yw; UAS-mCD8::GFP//; Mef2-Gal4//* TO *Oregon-R-C*

Fig. S5B-C	unpg labeling	Stock *w ; TI{GFP[3xP3.cLa]=CRIMIC.TG4.1}unpg[CR00787-TG4.1], UAS-mCD8::GFP, Mhc-RFP/CyO ; MKRS/TM6b*
Fig. S5D-E	tnc labeling	Stock *y, w, hs-Flp1.22; UAS-mCD8::GFP, Mhc-RFP/CyO; PBac{w[+mC]=IT.GAL4}2090-G4/TM6b*
Fig. S6A	tnc gal80ts and Mhc labeling	*y, w, hs-Flp1.22; UAS-mCD8::GFP, Mhc-RFP/CyO; PBac{w[+mC]=IT.GAL4}2090-G4/TM6b*<br>TO *P{w[+mC]=tubP-GAL80[ts]}Sxl[9], w /FM7c;+;+*
Fig. S6B-C	tnc labeling	Stock *y, w, hs-Flp1.22; UAS-mCD8::GFP, Mhc-RFP/CyO; PBac{w[+mC]=IT.GAL4}2090-G4/TM6b*
Fig. S6F	bi labeling	*y, w, P{w[+m*]=GAL4}MD735;+;+*<br>TO *y, w, hs-Flp1.22; UAS-mCD8::GFP/CyO; TM6b/MKRS*
Fig. S6G-H	tilm labeling	Stock *y, w, hs-Flp1.22; PBac{w[+mC]=IT.GAL4}RtGEF[4046-G4], UAS-mCD8::GFP, Mhc-RFP/CyO; MKRS/TM6b*
Fig. S7A	Control RNAi bi	Stock *y, w; Mhc-GAL4, UAS-mCD8::GFP, Mhc-RFP/CyO; zfh1-T2A-GAL4, UAS-Dcr-2 / TM6b*
Fig. S7B	RNAi bi KK108384	*y, w; Mhc-GAL4, UAS-mCD8::GFP,Mhc-RFP/CyO; zfh1-T2A-GAL4, UAS-Dcr-2/TM6b*<br>TO *P{KK108384}VIE-260B*
Fig. S7C	Control RNAi Lim1	Stock *y, w; Mhc-GAL4, UAS-mCD8::GFP,Mhc-RFP/CyO; zfh1-T2A-GAL4, UAS-Dcr-2/TM6b*
Fig. S7D	RNAi Lim1 KK	*y, w; Mhc-GAL4, UAS-mCD8::GFP,Mhc-RFP/CyO; zfh1-T2A-GAL4, UAS-Dcr-2/TM6b*<br>TO *P{KK109046}VIE-260B*
Fig. S7E	RNAi Lim1 TRiP	*y, w; Mhc-GAL4, UAS-mCD8::GFP,Mhc-RFP/CyO; zfh1-T2A-GAL4, UAS-Dcr-2/TM6b*<br>TO *P{y[+t7.7] v[+t1.8]=TRiP.JF02503}attP2*
Fig. S7F	RNAi Lim1TRiP + KK	*y, w; Mhc-GAL4, UAS-mCD8::GFP,Mhc-RFP/CyO; zfh1-T2A-GAL4, UAS-Dcr-2/TM6b*<br>TO *y, w ; P{KK109046}VIE-260B/CyO ; P{y[+t7.7] v[+t1.8]=TRiP.JF02503}attP2/TM6b*
Fig. S7G	Control RNAi Dr	Stock *y, w; Mhc-GAL4, UAS-mCD8::GFP,Mhc-RFP/CyO; zfh1-T2A-GAL4, UAS-Dcr-2/TM6b*
Fig. S7H	RNAi Dr	*y, w; Mhc-GAL4, UAS-mCD8::GFP,Mhc-RFP/CyO; zfh1-T2A-GAL4, UAS-Dcr-2/TM6b*<br>TO *P{KK107967}VIE-260B*

Fig. S8A	Zfh1 labeling	Stock *y, w ; zfh1-T2A-GAL4, UAS-mCD8::GFP/TM6b*
Fig. S8B	Control RNAi	Stock *y, w; Mhc-GAL4, UAS-mCD8::GFP,Mhc-RFP/CyO; zfh1-T2A-GAL4, UAS-Dcr-2/TM6b*
Fig. S8C	RNAi Lim1	*y, w; Mhc-GAL4, UAS-mCD8::GFP,Mhc-RFP/CyO; zfh1-T2A-GAL4, UAS-Dcr-2/TM6b*<br>TO *y, w ; P{KK109046}VIE-260B/CyO ; P{y[+t7.7] v[+t1.8]=TRiP.JF02503}attP2/TM6b*
Fig. S8D	RNAi bi	*y, w; Mhc-GAL4, UAS-mCD8::GFP,Mhc-RFP/CyO; zfh1-T2A-GAL4, UAS-Dcr-2/TM6b*<br>TO *P{KK108384}VIE-260B*
Fig. S10 A	control wg, unpg readout	*W ; wg[l-12],cn[1],UAS-mCD8::GFP/CyO, Tb;+*<br>TO *w; wg[l-12], TI{GFP[3xP3.cLa]=CRIMIC.TG4.1}unpg[CR00787-TG4.1]/CyO, bw, Tb;+*
Fig. S10 B	mutant wg, unpg readout	*w; wg[l-12],cn[1],UAS-mCD8::GFP/CyO, Tb;+*<br>TO *w; wg[l-12], TI{GFP[3xP3.cLa]=CRIMIC.TG4.1}unpg[CR00787-TG4.1]/CyO, bw, Tb;+*

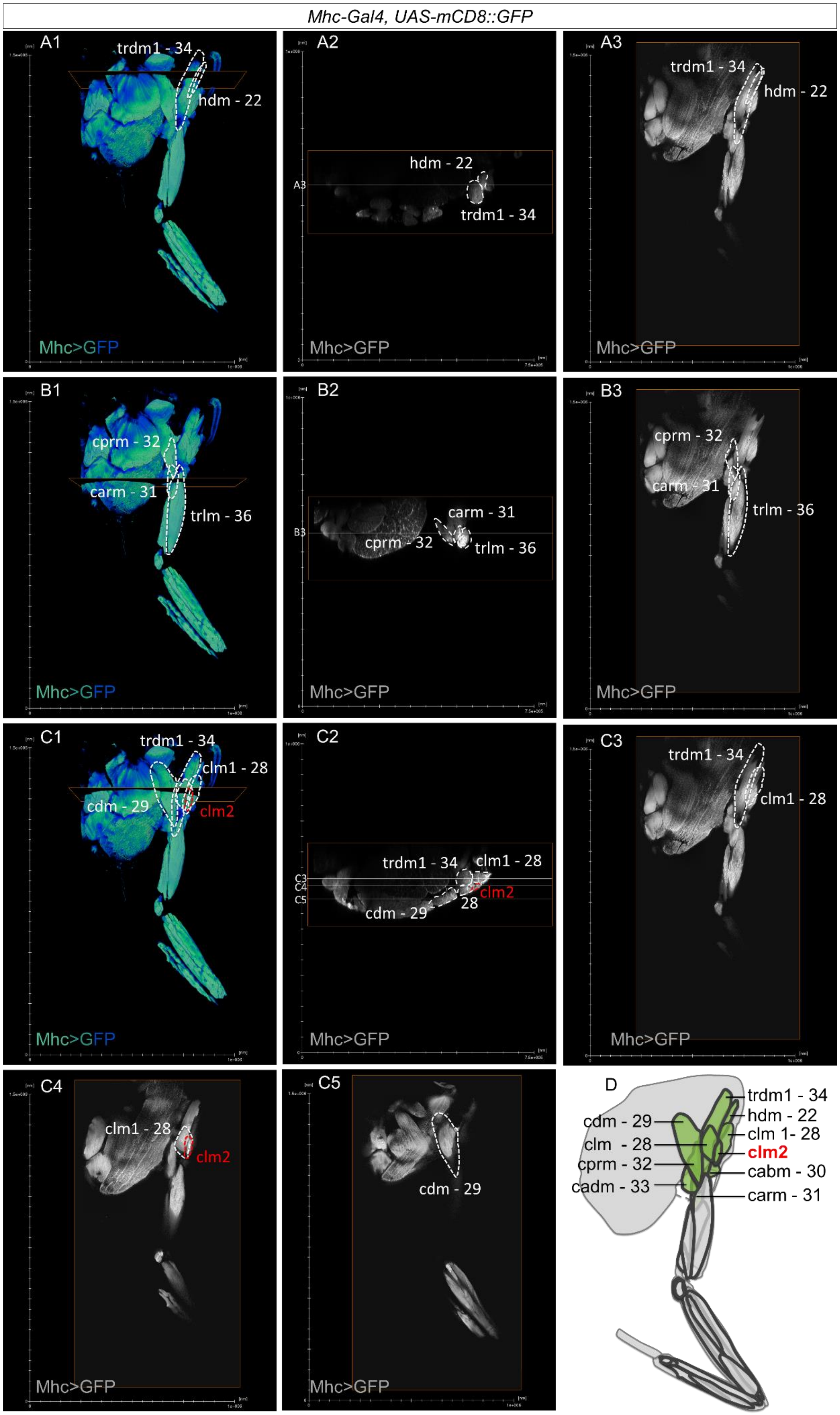

Mhc-Gal4, UAS-mCD8::GFP
A1
trdm1 - 34
hdm - 22
Mhc>GFP
A2
hdm - 22
trdm1 - 34
A3
Mhc>GFP
A3
trdm1 - 34
hdm - 22
Mhc>GFP
B1
cprm - 32
carm - 31
trlm - 36
Mhc>GFP
B2
carm - 31
B3
cprm - 32
trlm - 36
Mhc>GFP
B3
cprm - 32
carm - 31
trlm - 36
Mhc>GFP
C1
trdm1 - 34
clm1 - 28
cdm - 29
clm2
Mhc>GFP
C2
trdm1 - 34
clm1 - 28
C3
C4
C5
clm2
28
cdm - 29
Mhc>GFP
C3
trdm1 - 34
clm1 - 28
Mhc>GFP
C4
clm1 - 28
clm2
Mhc>GFP
C5
cdm - 29
Mhc>GFP
D
cdm - 29
clm - 28
cprm - 32
cadm - 33
trdm1 - 34
hdm - 22
clm 1- 28
clm2
cabm - 30
carm - 31

Fig. S1: Description of the prothoracic muscles.

(A1, B1, C1) 3D renderings of confocal sections showing the prothorax and the attached T1 leg, with muscles genetically labeled using *Mhc-GAL4>UAS-mCD8::GFP*.

(A2, B2, C2) Transverse sections; the positions of these sections are indicated in **(A1, B1, C1)**. These three transverse views were selected to highlight the positions of prothoracic muscles, outlined by dotted lines. A previously undescribed muscle, the coxa levator muscle 2 (***clm2***), is shown in red.

(A3, B3, C3, C4, C5) Sagittal sections; their positions are indicated by white lines in **(A2, B2, C2)**. These sections highlight the positions of the prothoracic muscles outlined with dotted lines. The muscle **clm2**, not described before, is marked in red.

(D) Schematic representation of all adult thoracic muscles. The prothoracic muscles described in this figure are shown in green.

Muscle nomenclature follows C. Soler and P.A. Lawrence article (*2*, *3*): m: muscle; hd: head dorsal; c: coxa; tr: trochanter; fe: femur; ti: tibia; ta: tarsus; l: levator; d: depressor; ab: abductor; ar: anterior rotator; pr: posterior rotator; ad: adductor; r: reductor; lt: long tendon or A. Miller's nomenclature (*59*): numbers.

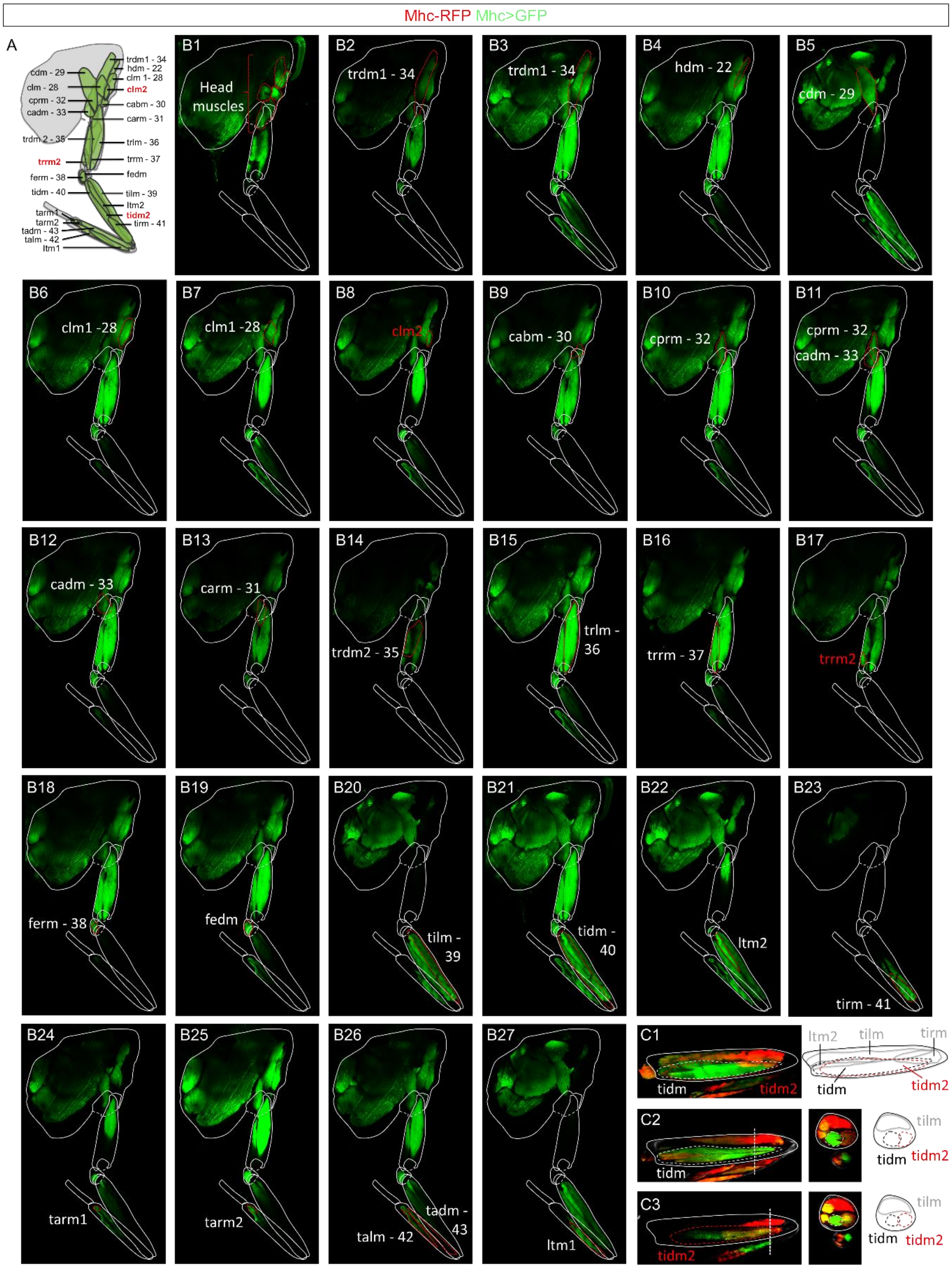

Mhc-RFP Mhc>GFP
A
cdm - 29
clm - 28
cprm - 32
cadm - 33
trdm 2 - 35
trrm2
ferm - 38
tidm - 40
tarm1
tarm2
tadm - 43
talm - 42
ltm1
trdm1 - 34
hdm - 22
clm 1- 28
clm2
cabm - 30
carm - 31
trlm - 36
trrm - 37
fedm
tilm - 39
ltm2
tidm2
tirm - 41
B1
Head muscles
B2
trdm1 - 34
B3
trdm1 - 34
B4
hdm - 22
B5
cdm - 29
B6
clm1 -28
B7
clm1 -28
B8
clm2
B9
cabm - 30
B10
cprm - 32
B11
cprm - 32
cadm - 33
B12
cadm - 33
B13
carm - 31
B14
trdm2 - 35
B15
trlm - 36
B16
trrm - 37
B17
trrm2
B18
ferm - 38
B19
fedm
B20
tilm - 39
B21
tidm - 40
B22
ltm2
B23
tirm - 41
B24
tarm1
B25
tarm2
B26
tadm - 43
talm - 42
B27
ltm1
C1
tidm
tidm2
ltm2
tilm
tirm
tidm
tidm2
C2
tidm
tilm
tidm
tidm2
C3
tidm2
tilm
tidm
tidm2

Fig. S2. Description of the leg muscles.

(A) Schematic representation of all adult thoracic T1 leg muscles. Muscle nomenclature follows C. Soler and P.A. Lawrence article (*2*, *3*): m: muscle; hd: head dorsal; c: coxa; tr: trochanter; fe: femur; ti: tibia; ta: tarsus; l: levator; d: depressor; ab: abductor; ar: anterior rotator; pr: posterior rotator; ad: adductor; r: reductor; lt: long tendon or A. Miller's nomenclature (*1*): numbers.

(B1–B27) confocal sections showing the prothorax and the attached T1 leg, with muscles genetically labeled using *Mhc-GAL4>UAS-mCD8::GFP*. These sections highlight muscles outlined with dotted lines.

(C1–C3) Adult femur of T1 legs genetically labeled with *Mhc-RFP* (red) and *UAS-mCD8::GFP* (green) under the control of *Mhc-GAL4*. **(C1)** shows a maximum projection; **(C2–C3)** are two confocal sections through the *tidm* or the deeper *tidm2*. To the left of **(C1)** is a schematic of the femoral muscles. To the right of **(C2–C3)** are two different cross-sections with their corresponding schematics. The positions of the cross-sections are indicated in **(C2–C3)** with dotted lines

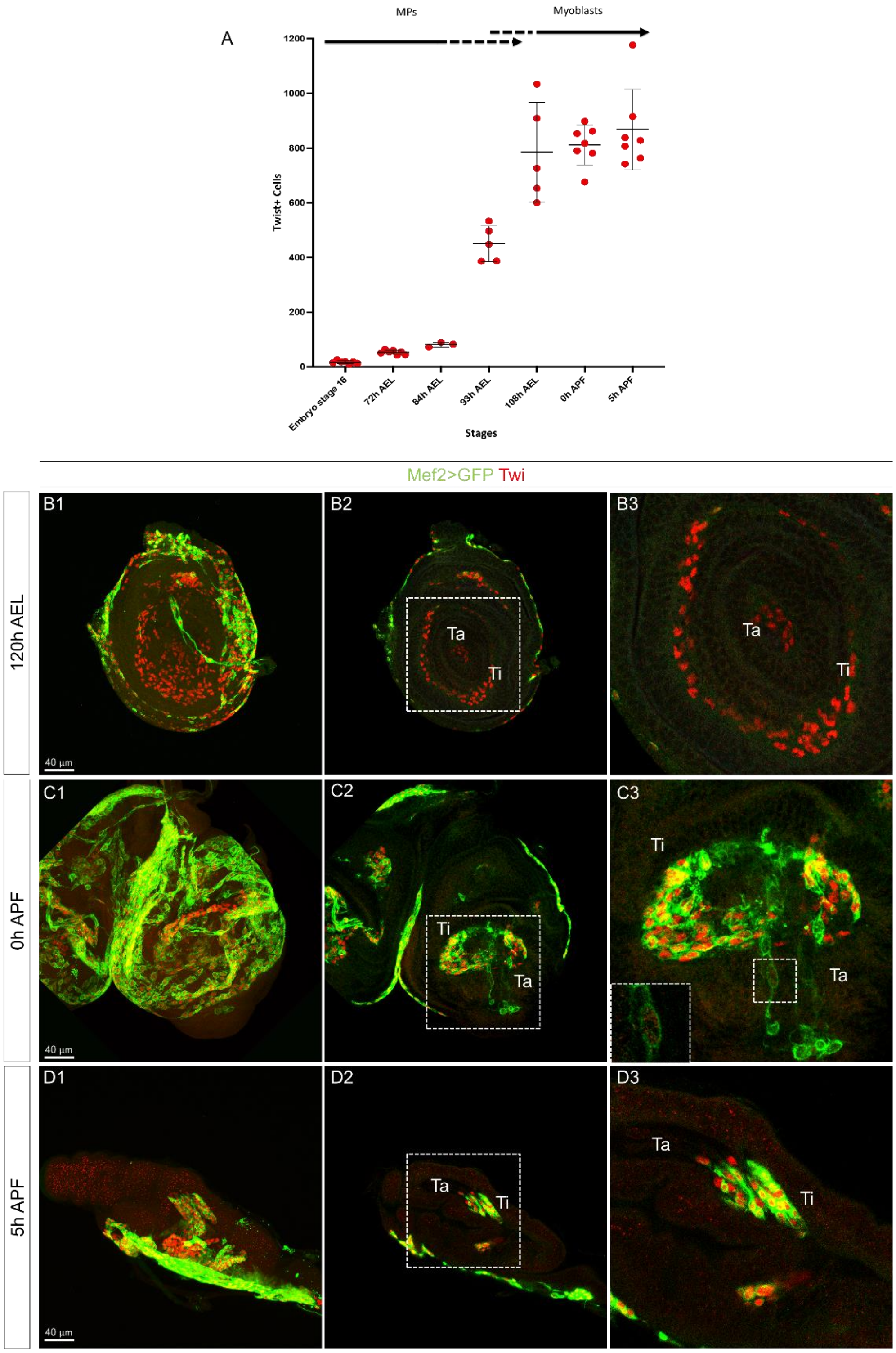
A
MPs
Myoblasts
1200
1000
800
600
400
200
0
Twist+ Cells
Embryo stage 16
72h AEL
84h AEL
93h AEL
108h AEL
0h APF
5h APF
Stages
Mef2>GFP Twi
120h AEL
0h APF
5h APF
B1
B2
B3
C1
C2
C3
D1
D2
D3
Ta
Ti
40 µm

Fig. S3. Dynamics of *Mef2* and Twist expression during late larval stages and early pupal stages.

(A) Graph of the number of Twi^+ cells during development.

(B1–D3) T1 leg discs immunostained for Twist (red) and genetically labeled with GFP (green) under the control of *Mef2-GAL4*. **(B1, C1, D1)** are maximum projections, **(B2, D2)** are confocal sections, and **(C2)** is a maximum projection of a few confocal sections. **(B3, C3, D3)** show enlargement of the dotted box in **(B2, C2, D2). (B3)** shows Twi^+ $Mef2^-$ cells in the tarsus, **(C3)** shows a weak Twi^+ cell and weak $Mef2>GFP^+$ cells in the tarsus, **(D3)** shows the absence of Twi^+ and $Mef2>GFP^+$ cells in the tarsus. **Note:** depending on the sample, weak $Mef2>GFP^+$ can be detected as shown in **(Fig. 2).** Ti: Tibia, Ta: Tarsus.

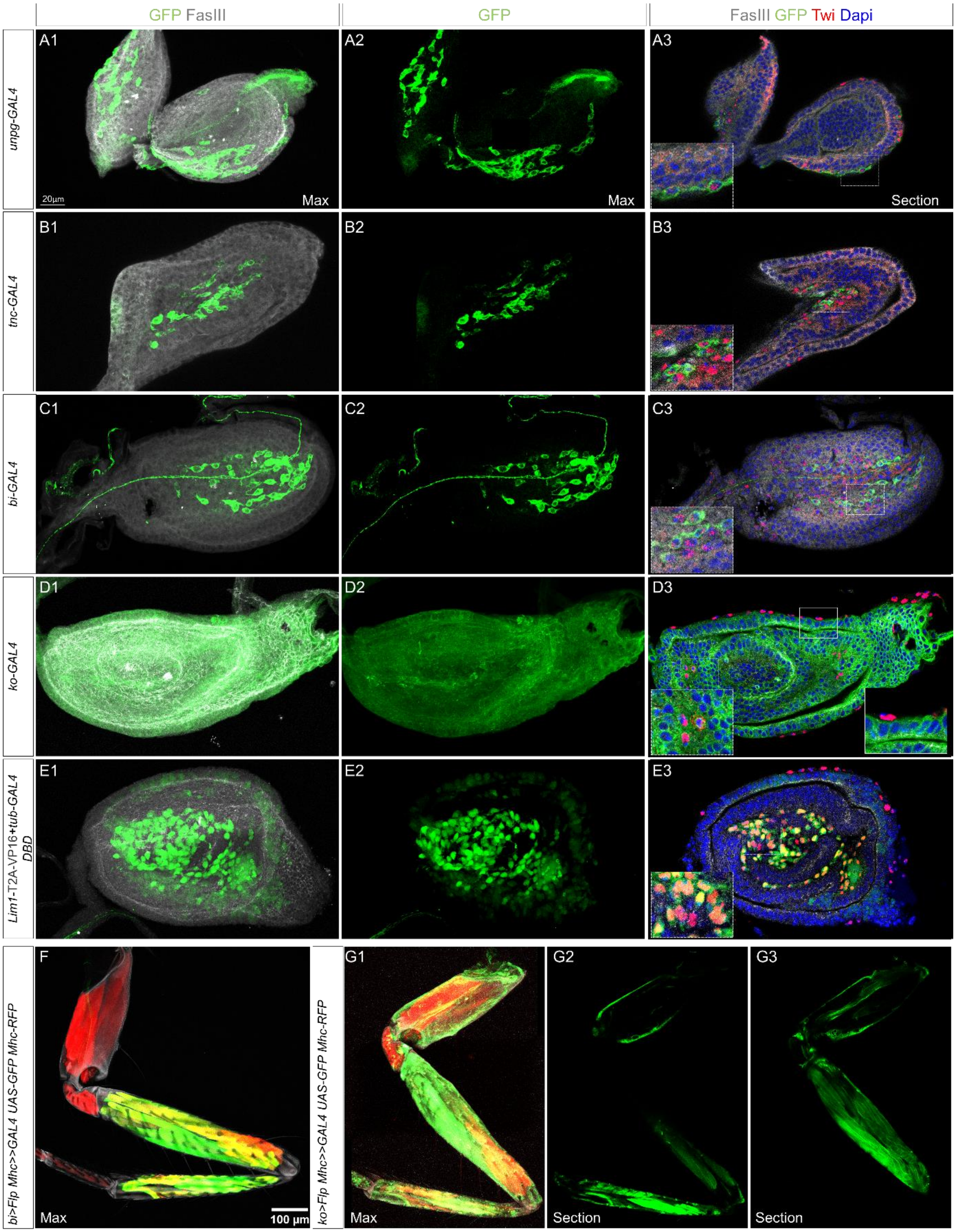

GFP FasIII
GFP
FasIII GFP Twi Dapi
unpg-GAL4
A1
20µm
Max
A2
Max
A3
Section
tnc-GAL4
B1
B2
B3
bi-GAL4
C1
C2
C3
ko-GAL4
D1
D2
D3
Lim1-T2A-VP16+tub-GAL4 DBD
E1
E2
E3
bi>Flp Mhc>>GAL4 UAS-GFP Mhc-RFP
F
Max
100 µm
ko>Flp Mhc>>GAL4 UAS-GFP Mhc-RFP
G1
Max
G2
Section
G3
Section

Fig. S4. Recovering the spatial information and determining the identity of the central and peripheral MP clusters.

(A1–E3) T1 leg discs at 96 hours AEL immunostained for FasIII (grey) and Twist (red), counterstained with DAPI (blue), and genetically labeled with GFP (green) under the control of *unpg-GAL4* **(A1–A3),** *tnc-GAL4* **(B1–B3),** *bi-GAL4* **(C1–C3),** *ko-GAL4* **(D1–D3),** and *Lim1-VP16AD+tub-GAL4DBD* **(E1–E3). (A1–E2)** show maximum projections; **(A3–E3)** are corresponding confocal sections. The bottom left dotted boxes in **(A3, B3, C3, D3, E3)** highlight GFP^{+} and Twi^{+} MPs; the bottom right box in **(D3)** highlights GFP^{-} but Twi^{+} MPs, showing the absence of Ko in peripheral MPs.

(F–G3) T1 legs labeled with *Mhc-RFP* (red) showing GFP expression in distal and proximal muscles following lineage tracing activated in MPs using *bi-GAL4* **(F)** or *ko-GAL4* **(G1–G3),** respectively. (**F, G1)** are maximum projections; **(G2, G3)** are confocal sections.

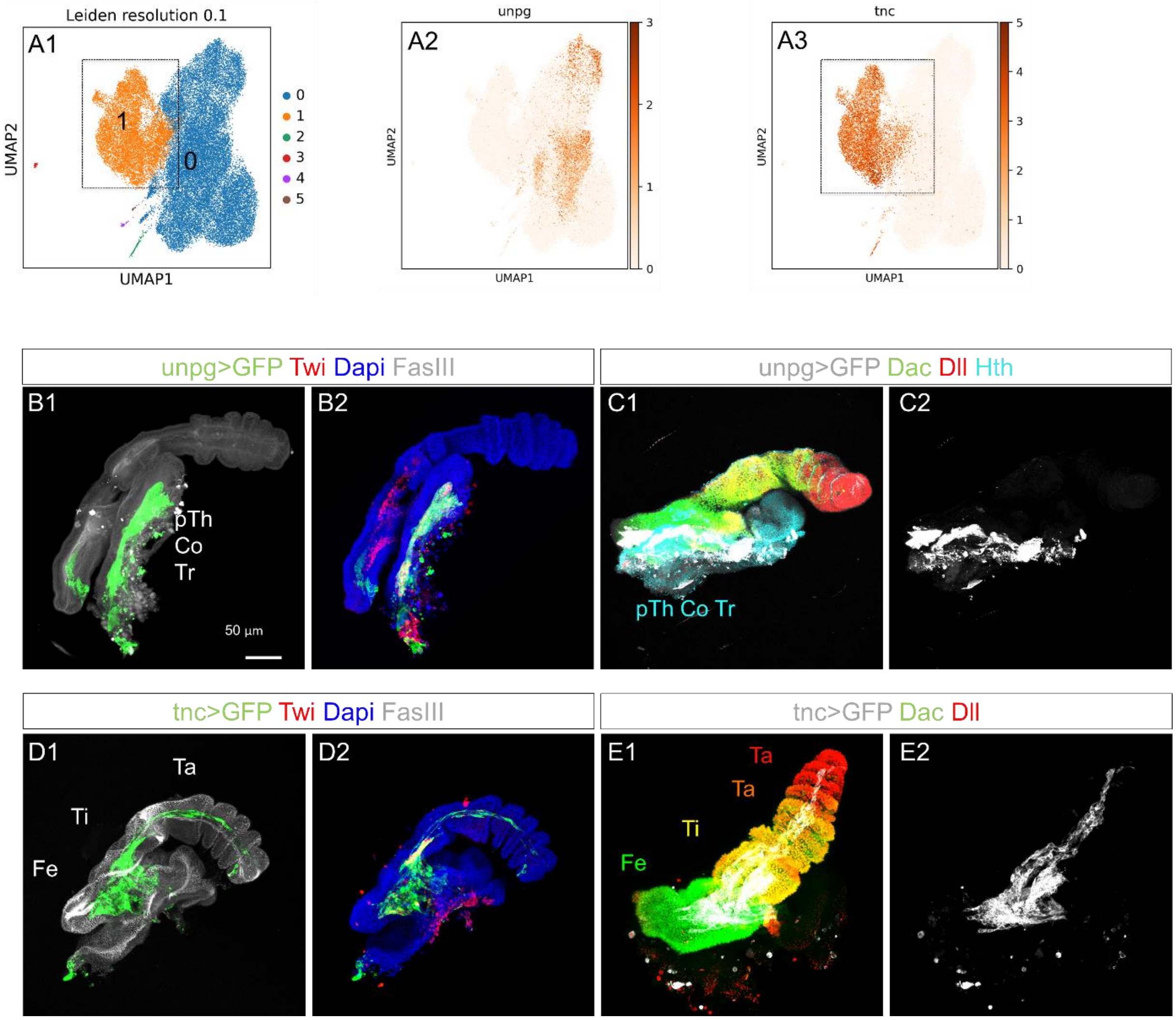

Fig. S5. MPs/myoblasts are spatially and transcriptionally subdivided into central and peripheral populations

(A1–A3) Low-resolution UMAP revealing two major clusters **(A1)** and the differential expression of *unpg* **(A2)** and *tnc* **(A3)** in these two clusters.

(B1–E2) T1 imaginal discs at 5 hours APF genetically labeled with *UAS-mCD8::GFP* (green or white) under the control of *unpg-GAL4* **(B1–C2)** or *tnc-GAL4* **(D1–E2),** immunostained with FasIII (grey), Twi (red), and DAPI (nuclei, blue) **(B1–B2, D1–D2),** or with Dac (green), Dll (red), and Hth (cyan) **(C1–C2, E1–E2)**. Tr: trochanter; Co: coxa; pTh: prothorax; Fe: femur; Ti: tibia; Ta: tarsus.

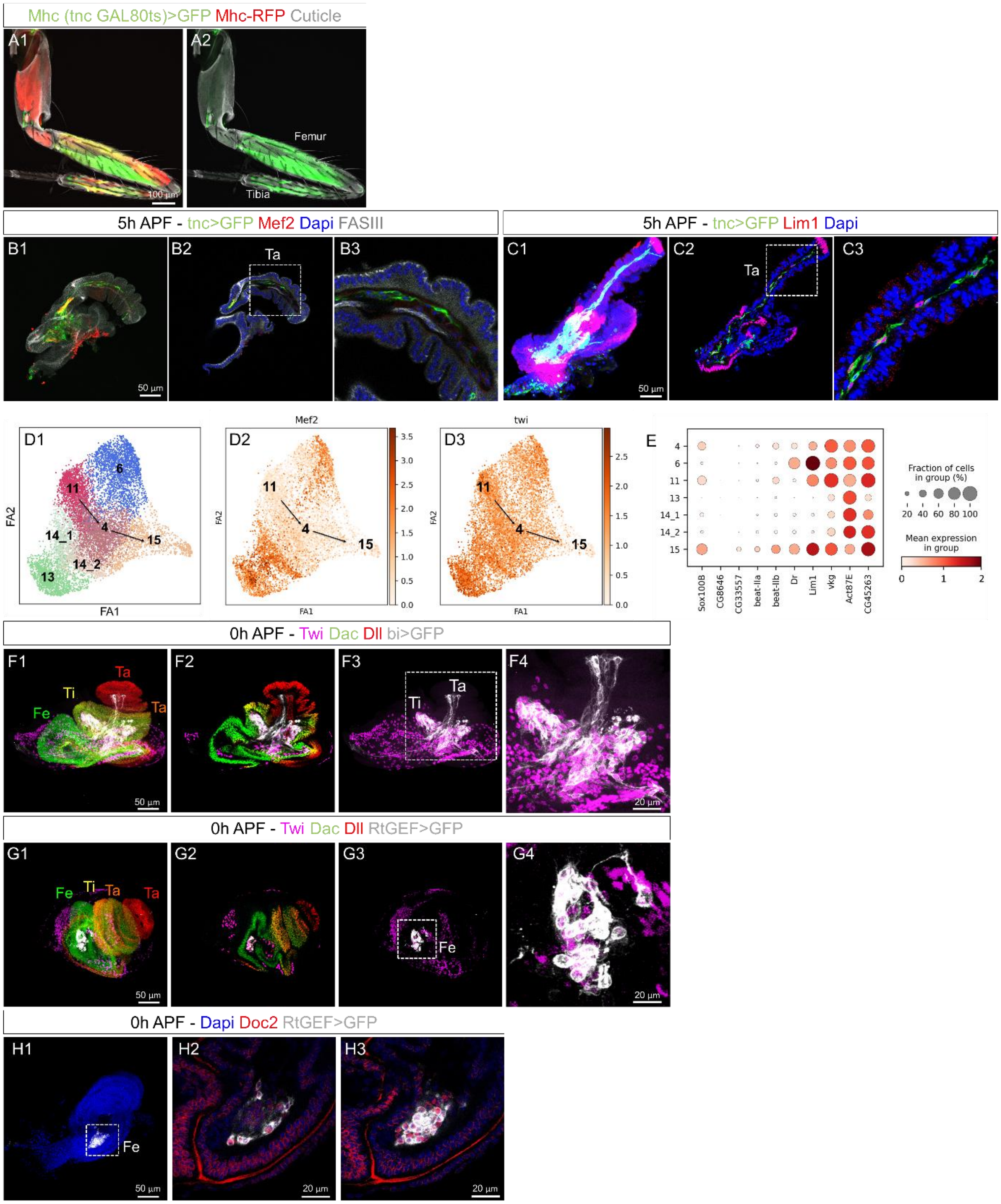

Mhc (tnc GAL80ts)>GFP Mhc-RFP Cuticle
A1
A2
Femur
Tibia
100 µm
5h APF - tnc>GFP Mef2 Dapi FASIII
B1
B2
B3
Ta
50 µm
5h APF - tnc>GFP Lim1 Dapi
C1
C2
C3
Ta
50 µm
D1
D2
D3
Mef2
twi
FA1
FA2
E
Fraction of cells in group (%)
20 40 60 80 100
Mean expression in group
0 1 2
Sox100B
CG8646
CG33557
beat-IIa
beat-IIb
Dr
Lim1
vkg
Act87E
CG45263
0h APF - Twi Dac Dll bi>GFP
F1
F2
F3
F4
Fe
Ti
Ta
50 µm
20 µm
0h APF - Twi Dac Dll RtGEF>GFP
G1
G2
G3
G4
0h APF - Dapi Doc2 RtGEF>GFP
H1
H2
H3

Fig. S6. Recovering the spatial information and determining the identity of myoblast clusters at pupal stages.

(A1–A2) T1 legs labeled with *Mhc-RFP* (red), showing GFP expression in distal and proximal muscles following lineage tracing activated in MPs using *tnc-GAL4*. *tubP-GAL80*^{ts} was used to activate lineage tracing only after 0 hour APF. Note: This late induction is sufficient to label all telopodite muscles, confirming that *tnc-GAL4* is a robust marker for telopodite lineages even at later stages.

(B1–B3) Immature T1 legs at 5 hours APF genetically labeled with *UAS-mCD8::GFP* (green) under the control of *tnc-GAL4,* immunostained for FasIII (grey) and Mef2 (red), counterstained with DAPI (blue). **(B1)** is a maximum projection; **(B2)** is a confocal section; the dotted box in **(B2)** is enlarged in (**B3**). Note: tnc>GFP^{+} Mef2^{-} cells are present in the tarsus **(B3)**, and also in the immature femur and tibia (not shown).

(C1–C3) Immature T1 legs at 5 hours APF genetically labeled with *UAS-mCD8::GFP* (green) under the control of *tnc-GAL4,* immunostained for Lim1 (red), counterstained with DAPI (blue). **(C1)** is a maximum projection; **(C2)** is a confocal section; the dotted box in **(C2)** is enlarged in **(C3)**. Note: tnc>GFP^{+} Lim1^{+} cells are present in the tarsus **(C3)**.

(D1–D3) FA2 map showing distinct central clusters. **(D1)** Arrows indicate the 11→4→15 lineage; **(D2-D3)** Expression patterns of Mef2 and Twi, respectively.

(E) Dot plot showing the expression of neuronal lamella markers. Note: The absence of Mef2 (**B1–B2, D2)**, inhibition of Twi **(D3 and see Fig. 3)**, and the expression of neuronal lamella markers (**E, C1-C3, and Fig. S3)** indicate that the 11→4→15 lineage gives rise to the neuronal lamella.

(F1–F4) Immature T1 legs at 0 hour APF expressing *bi>GFP* (grey), immunostained for Dll (red), Dac (green), and Twi (purple). **(F1)** is a maximum projection; **(F2)** is a confocal section; **(F3)** is a maximum projection; **(F4)** shows an enlargement of the dotted box in **(F3)**. Note: bi is a good marker for cluster 14_2 and weakly expressed in cluster 15 **(see Fig. 5G1-H4)**. GFP expression confirms low levels in Twi^{-} cells in the tarsus (Ta), tibia (Ti), and femur (Fe), and high expression in Twi^{+} cells in the tibia (Ti).

(G1–G4) Immature T1 legs at 0 hour APF expressing *RtGEF>GFP* (grey), immunostained for Dll (red), Dac (green), and Twi (purple). **(G1)** is a maximum projection; **(G2)** is a confocal section; **(G3)** is a maximum projection; **(G4)** shows an enlargement of the dotted box in **(G3)**. Note: *RtGEF>GFP* is a specific marker for cluster 6, which is localized in the dorsal femur (Fe).

(H1–H3) Immature T1 legs at 0 hour APF expressing *RtGEF>GFP* (grey), immunostained for Doc2 (a specific marker of cluster 6, red), and counterstained with DAPI (blue). **(H1)** is a maximum projection; **(H2–H3)** are two different confocal sections showing enlargements of the dotted box in **(H1)**.

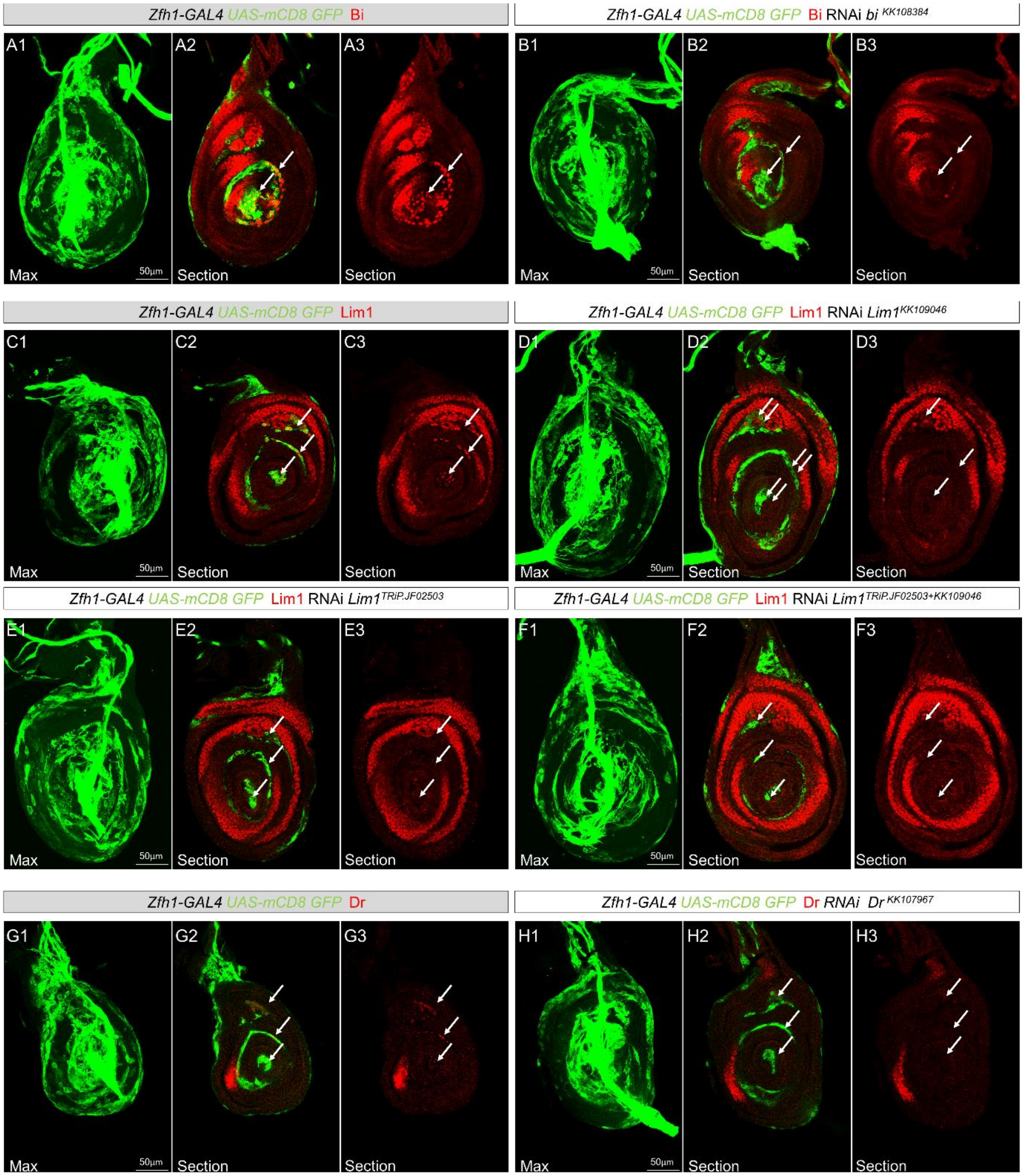

Fig. S7. Assessment of RNAi efficiency against *Bi*, *Lim1*, and *Dr*.

(A1–H3) Late third instar T1 leg disc genetically labeled with *zfh1-GAL4>UAS-mCD8::GFP* (green) and immunostained for Bi (red) **(A1–B3)** or Lim1 (red) **(C1–F3)** or Dr (red) **(G1–H3)** in control flies **(A1–A3, C1–C3, G1–G3)**, or following RNAi-mediated knockdown of *bi* **(B1–B3)**, *Lim1* simple KD **(D1–D3, E1-E3)**, *Lim1* double KD **(F1–F3)**, or *Dr* **(H1–H3)** flies. White arrows indicate Bi+ (red) **(A2–A3)** or Lim+ (red) **(C2–C3)** or Dr+ **(G2–3)** myoblasts in control flies or their absence in RNAi-mediated knockdown **(B2–B3, D2–D3, E2-E3, F2–F3, H2–H3).**

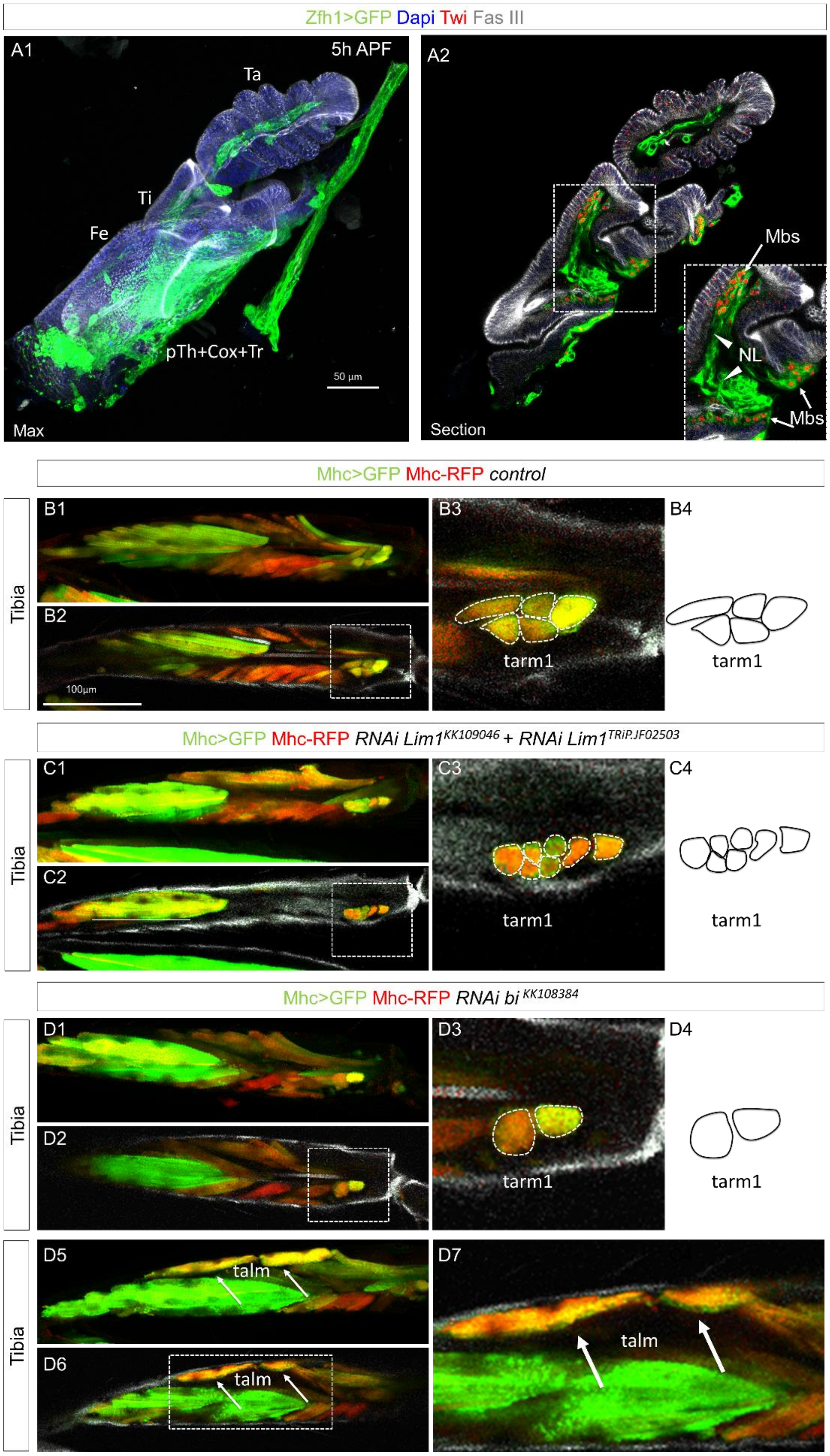

Zfh1>GFP Dapi Twi Fas III
A1
5h APF
Ta
Ti
Fe
pTh+Cox+Tr
50 µm
Max
A2
Mbs
NL
Mbs
Section
Mhc>GFP Mhc-RFP control
Tibia
B1
B2
100µm
B3
tarm1
B4
tarm1
Mhc>GFP Mhc-RFP RNAi Lim1KK109046 + RNAi Lim1TRiP.JF02503
Tibia
C1
C2
C3
tarm1
C4
tarm1
Mhc>GFP Mhc-RFP RNAi bi KK108384
Tibia
D1
D2
D3
tarm1
D4
tarm1
Tibia
D5
talm
D6
talm
D7
talm

Fig. S8. Expression of *Zfh1-GAL4* in immature legs and the effects of *bi* and *lim1* RNAi on muscle architecture using the *Zfh1-GAL4* driver.

(A1–A2) Immature legs at 5 hours APF genetically labeled with *Zfh1-GAL4>UAS-mCD8::GFP* (green) and immunostained for Twist (Twi, red) and FasIII (gray), with nuclei labeled by DAPI (blue). **(A1)** shows a maximum projection and **(A2)** a single confocal section. The dotted box in **(A2)** highlights Zfh1>GFP+ myoblasts (Mbs, arrows) expressing Twist. Zfh1>GFP+ and Twi– cells correspond to neuronal lamella cells (NL, arrowhead). pTh: prothorax, Cox: coxa, Tr: trochanter, Fe: femur, Ti: tibia, Ta: tarses.

(B1–D7) Adult tibia of T1 legs genetically labeled with *Mhc-RFP* (red) and *UAS-mCD8::GFP* (green) under the control of *Mhc-GAL4*. Panels show: a control fly **(B1–B4)**, a fly with *Lim1* knockdown (KD) in MPs, myoblasts, and muscles **(C1–C4)**, and a fly with *bi* KD in MPs, myoblasts, and muscles **(D1–D7)**. **(B1, C1, D, D5)** are maximum projections; **(B2, C2, D2, D6)** are confocal sections; **(B3, C3, D3, D7)** are enlargements of the dotted boxes in **(B2, C2, D2, D6)**; and **(B4, C4, D4)** are schematics of the tarsal muscles.

Note: In **(B1–D4)**, the number of tarsal muscle fibers is reduced in flies with *bi* KD **(see also Table S1)** and increased in flies with *lim1* KD. In **(D5–D7)**, rounded fibers are observed in tarsal muscles of flies with *bi* KD (arrows).

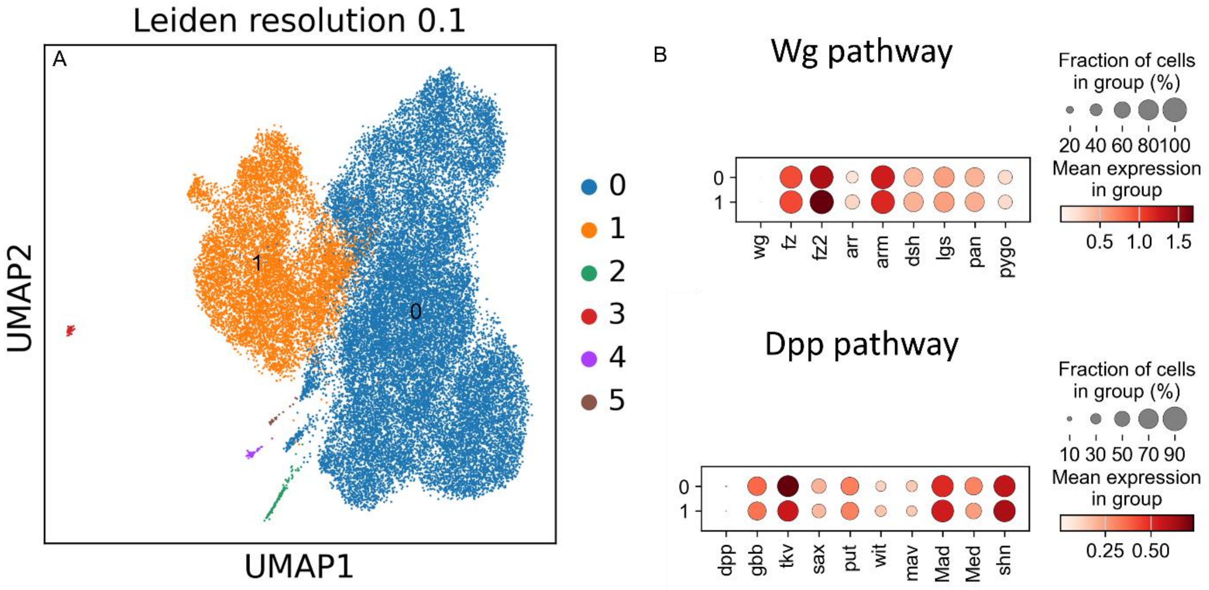

Fig. S9. MPs and myoblasts express all components required for Wg/Dpp signal reception and transduction but do not express the morphogens themselves.

(A) Low-resolution (0.1) of merged UMAP of all time points, revealing two major clusters.

(B) Dot plot showing the expression of Wg and Dpp, as well as the proteins involved in signal reception and transduction.

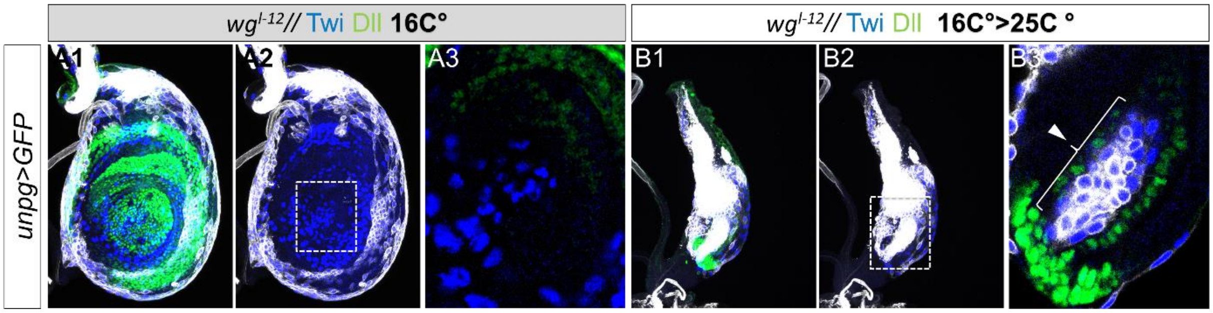

Fig. S10. Wg and Dpp secreted by the epithelium act synergistically to specify the central vs peripheral identity of MPs.

(A1–B3) Third instar leg discs of *wg*$^{1\text{-}12}$// mutants expressing *unpg-GAL4>UAS-mCD8::GFP* (gray). Discs were immunostained for Dll (green) and Twi (blue). Discs were maintained at the permissive temperature **(A1–A3)** or shifted to the non-permissive temperature shortly before the specification of central *vs* peripheral MPs **(B1–B3)**. Note: all central unpg>GFP^{+} cells are Twi^{+} as highlighted in zoom in **(A3)** and **(**head-arrow, **B3)**

Muscle name		Phenotypes observed in bi KD condition				
Tibia	tarm 1	Absent N=1	Fiber number reduced N=7			WT N=8
	tarm 2	Absent N=2				WT N=14
	tadm		Fiber number reduced N=15			WT N=1
	talm		Fiber number reduced N=2		Fibers attached to the wrong tendon or not attached N=12	WT N=2
	ltm1					WT N=16
Femur	tirm					WT N=16
	tidm					WT N=16
	tidm2					WT N=16
	tilm					WT N=16
	ltm2			Rounded fibers N=9		WT N=7

Table S1: Quantification of leg phenotypes observed in *bi* KD condition.

Movie S1. 3D segmentation of adult T1 leg muscles.

Movie S2. 3D segmentation of T1 leg myoblasts and neuronal lamella cells at 0 hour APF and 5 hours APF.

Tab S1. Quantification of muscle phenotype in bi KD condition.
